# Storage-Time-Aware Chemometric Deep Learning Model for Predicting Phenolic Retention and Antioxidant Capacity in Kefir Enriched with Alginate-Encapsulated Grape Seed Extract

**DOI:** 10.3390/foods15162858

**Published:** 2026-08-16

**Authors:** Özge Duygu Okur, Aytaç Altan

**Affiliations:** 1 Department of Food Engineering, Faculty of Engineering, Zonguldak Bülent Ecevit University, 67100 Zonguldak, Turkey; oduyguokur@beun.edu.tr; 2 Department of Electrical Electronics Engineering, Faculty of Engineering, Zonguldak Bülent Ecevit University, 67100 Zonguldak, Turkey

**Keywords:** kefir, black grape seed extract, alginate encapsulation, phenolic retention, antioxidant capacity, chemometric deep learning, crested porcupine optimizer

## Abstract

Kefir offers a suitable fermented dairy matrix for delivering plant-derived bioactives, yet maintaining and predicting phenolic compounds during refrigerated storage remains a technological challenge. This study developed a storage-time-aware chemometric deep learning model to estimate phenolic retention and antioxidant capacity in kefir enriched with free and alginate-encapsulated black grape seed extract. Five formulations were prepared, including control kefir, kefir containing 1% and 3% free extract, and kefir containing 1% and 3% alginate-encapsulated extract. Samples were stored at 4 °C and analyzed on Days 1, 7, and 14 for physicochemical, textural, color and bioactive properties. Total phenolic content and Trolox-equivalent antioxidant capacity were used as target responses, while ten quality descriptors were transformed into latent chemometric features and arranged as storage-time sequences. A convolutional recurrent deep learning architecture was then optimized using the crested porcupine optimizer (CPO) and compared with baseline and ablation models. Alginate encapsulation produced structurally intact beads with high encapsulation efficiency, supporting improved phenolic retention during storage. The proposed model provided the most accurate dual prediction of total phenolic content and antioxidant capacity. It outperformed non-optimized and partially ablated alternatives in terms of reducing error and achieving agreement between the observed and predicted values. Residual, normality and Bland–Altman analyses further supported the reliability of the prediction behavior. Overall, the results demonstrate that integrating alginate encapsulation with storage-time-aware chemometric deep learning provides a practical strategy for monitoring bioactive stability in functional kefir and offers a reproducible modelling framework for fermented dairy products enriched with phenolic-rich by-products.

## 1. Introduction

The growing interest in foods that provide benefits beyond basic nutrition has reshaped the development of fermented dairy products. Consumers increasingly seek products that support digestive health, contribute to immune balance, and help counteract oxidative stress through naturally derived bioactive compounds. In this context, fermented dairy matrices have become attractive carriers for functional ingredients because they combine nutritional value, microbial activity, and consumer familiarity [1,2]. Kefir occupies a distinct position among fermented dairy products. Its fermentation is driven by a complex symbiotic consortium of lactic acid bacteria, acetic acid bacteria, and yeasts, resulting in a matrix rich in organic acids, bioactive peptides, vitamins, exopolysaccharides, and antimicrobial metabolites. These compounds contribute to the antioxidant, antimicrobial, anti-inflammatory, immunomodulatory, and gastrointestinal effects commonly associated with kefir, making it a promising vehicle for plant-derived functional ingredients [3].

The functional profile of kefir can be further improved by enrichment with phenolic-rich plant materials. Fruits, herbs, edible flowers, and agro-industrial by-products have been incorporated into fermented dairy systems to enhance antioxidant capacity, phenolic composition, and nutritional value [4,5]. However, the effectiveness of such enrichment depends not only on the initial bioactive content of the added material but also on its behavior within the dairy matrix. During fermentation and refrigerated storage, phenolic compounds may undergo oxidation, interact with milk proteins, or exhibit formulation-dependent release and retention patterns. Therefore, the development of functional kefir requires strategies that preserve bioactive compounds and allow their changes during storage to be evaluated in a systematic manner.

Grape seeds are a particularly valuable source of phenolic compounds and represent an important by-product of the wine and grape juice industry. Their valorization is consistent with circular economy principles because it transforms an agricultural residue into a high-value ingredient for functional food development [6,7]. Black grape seed (*Vitis vinifera* L.) contains proanthocyanidins, catechins, epicatechins, gallic acid, and other flavonoids with strong antioxidant potential and reported biological effects, including anti-inflammatory, antimicrobial, cardioprotective, and anti-diabetic activities [7,8]. These properties make grape seed extract a promising ingredient for dairy fortification. Nevertheless, direct incorporation of free grape seed extract into fermented dairy products may not fully preserve its functional value. Polyphenols can be sensitive to acidic conditions, oxygen exposure, processing stress, and storage time. They may also form complexes with milk proteins, which can alter their bioaccessibility, technological behavior, and apparent antioxidant response [9,10].

Encapsulation has emerged as an effective approach for improving the stability and controlled delivery of sensitive bioactive compounds in food systems. Among different wall materials, sodium alginate is widely used because it is biocompatible, non-toxic, biodegradable, and able to form gel beads through ionic cross-linking under mild conditions [11,12,13,14]. Alginate-based encapsulation can reduce the exposure of phenolics to adverse environmental conditions, support their retention during storage, and regulate their release within food matrices or under digestive conditions [15,16]. For this reason, alginate beads containing grape seed extract offer a technologically relevant route for developing antioxidant-rich kefir. However, evaluating such systems requires more than measuring phenolic content at isolated time points. The key question is how formulation type, encapsulation status, physicochemical properties, texture, color, and storage time jointly influence phenolic retention and antioxidant capacity.

Conventional laboratory assays remain essential for quantifying total phenolic content and antioxidant capacity, but they have limitations when repeated monitoring is required. They are typically destructive, reagent-dependent, labor-intensive, and unsuitable for rapid prediction of storage-related changes. This limitation is especially relevant for functional kefir, where the response of bioactive compounds is not governed by a single variable. Instead, phenolic retention and antioxidant capacity emerge from a multivariate system involving acidity, water-holding capacity, textural structure, color attributes, formulation composition, and storage duration. A predictive framework capable of learning such combined effects could therefore support quality monitoring, formulation optimization, and reduction of repeated chemical analyses.

Artificial intelligence, machine learning, and deep learning have increasingly been used to model complex relationships in food systems. Classical machine learning approaches can capture nonlinear associations between quality descriptors and target responses, but their performance may be constrained when the dataset contains correlated predictors, limited sample size, multiple outputs, and time-dependent changes. Deep learning offers a more flexible framework by learning hierarchical representations from structured data [17]. Convolutional operations are useful for extracting local patterns from ordered inputs, whereas recurrent architectures, particularly long short-term memory networks, are designed to preserve information across sequential observations [18]. These characteristics make deep learning attractive for storage-related food quality prediction. However, direct application of deep architectures to small experimental food datasets may lead to overfitting, unstable training, and sensitivity to hyperparameter selection.

Hybrid modelling can help address these challenges by combining the interpretability of chemometrics with the representational capacity of deep learning. Principal component analysis is one of the most established chemometric tools for converting correlated physicochemical variables into orthogonal latent descriptors [19]. In fermented dairy datasets, where texture, color, acidity, and water-holding capacity may be interrelated, this transformation reduces redundancy while retaining the dominant variance structure. When principal component scores are organized according to storage day, they can form compact temporal descriptors of product evolution. A storage-time-aware model can then learn not only the relationship between formulation and bioactive response but also the trajectory of quality changes over time. In addition, optimizer-guided hyperparameter tuning can reduce the dependence on manual model configuration and improve the robustness of deep learning models trained on small experimental datasets.

Despite the increasing interest in functional dairy enrichment and data-driven food quality prediction, several gaps remain. Studies on grape seed-enriched fermented dairy products have mostly focused on experimental characterization, whereas predictive modelling of phenolic retention and antioxidant capacity during storage remains limited. Moreover, the comparison of free and alginate-encapsulated black grape seed extract in kefir has not been sufficiently linked to a storage-time-aware prediction framework. Many modelling approaches still treat each observation as an independent tabular record, thereby ignoring the ordered structure of storage days. These limitations indicate the need for an integrated experimental and computational framework that connects alginate-based delivery, storage-dependent bioactive behavior, chemometric feature transformation, temporal deep learning, and optimizer-guided model tuning.

To address these gaps, this study proposes a storage-time-aware chemometric deep learning model for predicting phenolic retention and antioxidant capacity in kefir enriched with alginate-encapsulated grape seed extract. Five kefir formulations were prepared: control kefir, kefir enriched with free black grape seed extract at two concentrations, and kefir enriched with alginate-encapsulated black grape seed extract at two concentrations. Samples were analyzed during refrigerated storage using physicochemical, textural, color, and bioactive measurements. The main contributions of this work are as follows:

- Black grape seed extract was valorized as a sustainable phenolic-rich ingredient for the development of functional kefir.
- Alginate encapsulation was used as a delivery strategy to support phenolic retention and antioxidant functionality during refrigerated storage.
- Free and alginate-encapsulated grape seed extract formulations were compared using physicochemical, textural, color, and bioactive quality descriptors across storage time.
- A storage-time-aware chemometric modelling structure was developed by converting correlated quality descriptors into latent features and arranging them as temporal storage sequences.
- A deep learning model was constructed to predict total phenolic content and antioxidant capacity simultaneously as dual bioactive responses.
- Optimizer-guided hyperparameter tuning was incorporated to improve model configuration and reduce dependence on manual trial-and-error selection.
- The predictive behavior of the model was evaluated using error metrics, ablation analysis, residual diagnostics, normality assessment, and agreement analysis.

Through this integrated design, the study aims to contribute both to the development of bioactive kefir formulations and to the broader use of reproducible prediction models for monitoring functional quality in fermented dairy products.

The remainder of this paper is organized as follows. Section 2 presents the background and related works on grape seed bioactives, encapsulation strategies, artificial intelligence-based food quality modelling, and hybrid chemometric deep learning approaches. Section 3 describes the materials and methods, including kefir formulation, alginate encapsulation, analytical measurements, data preprocessing, storage-time sequence construction, model architecture, optimizer-guided hyperparameter tuning, and validation procedures. Section 4 presents the results and discussion, including experimental characterization, chemometric interpretation, model performance, ablation findings, and diagnostic analyses. Section 5 concludes the study by summarizing the main findings, methodological implications, limitations, and future research directions.

## 2. Background and Related Works

The use of data-driven tools in fermented dairy research has gradually moved from simple process description toward prediction, monitoring, and decision support. This shift is mainly related to the nature of fermentation itself. In kefir and yoghurt systems, product quality is shaped by several variables acting together, including incubation temperature, fermentation time, inoculum level, pH development, microbial activity, storage period, and matrix composition. Since these variables do not act independently, conventional endpoint measurements may describe the final product but cannot fully explain or predict the route by which quality changes occur.

Kefir-specific artificial intelligence studies are still limited, and most of them have focused on fuzzy logic rather than deep learning. Akgül et al. [20] modelled kefir production using incubation temperature, incubation time, and culture inoculum ratio as input variables, with the practical aim of supporting the control of fermentation toward the desired pH endpoint. Pacco [21] later used Mamdani fuzzy logic in LabVIEW to simulate and control kefir fermentation, again emphasizing time, temperature, and pH as the main control variables. Akıllı et al. [22] extended this line of work from process control to quality classification by developing a fuzzy logic-based decision support system for kefir samples. Their system used incubation temperature, incubation time, storage time, total bacterial count, and pH, and reported strong agreement with expert decisions. These studies are important because they show that kefir quality can be handled by intelligent models; however, they do not address phenolic retention, antioxidant capacity, encapsulated plant extracts, or storage-time-based deep prediction.

In parallel, yoghurt and other fermented dairy products have been used more frequently as test systems for machine learning and non-destructive monitoring. Ray et al. [23] developed a real-time yoghurt fermentation monitoring system using electrical conductivity, pH, temperature, and total dissolved solids, and compared several machine learning and deep learning models for pH prediction. Saetae [24] combined long short-term memory networks with classical microbial growth models and support vector regression to estimate microbial growth and acidification under industrial fermentation temperatures. Other studies have taken a different route by using optical or visual information. Alvarado et al. [25] predicted pH and titratable acidity during yoghurt fermentation from CIELAB color parameters using artificial neural networks, while Chopde et al. [26] showed that selected color indices extracted from yoghurt images could follow pH changes during fermentation. Allende-Prieto et al. [27] used visible and near-infrared spectroscopy with multivariate modelling to estimate pH, viscosity, and milk origin in yoghurt. These studies support the idea that dairy quality attributes can be estimated from indirect descriptors, but their focus is mainly fermentation progress rather than storage-dependent bioactive quality.

For functional fermented beverages, chemometric modelling provides another useful background. Araújo et al. [28] used mid-infrared spectroscopy with principal component analysis and partial least squares regression to predict phenolic compounds, antioxidant activity, and anthocyanins in a non-dairy water kefir beverage. This study is especially relevant because it links kefir fermentation with bioactive compound prediction. Nevertheless, it remains a spectroscopic calibration study and does not use a deep temporal model or an encapsulated phenolic delivery system. More recent smart-fermentation studies also indicate that sensor data, digital platforms, and machine learning can be combined for acidity control in fermented dairy products. For example, Adeleke et al. [29] integrated internet of things (IoT)-based sensing, machine learning, digital twin visualization, and control logic for amasi fermentation. At a broader level, Asar et al. [30] discussed the expanding role of artificial intelligence in probiotic research, including viability prediction, technological optimization, storage stability, and functional characterization.

Taken together, the literature shows that artificial intelligence has already been introduced into kefir production, dairy fermentation monitoring, and chemometric prediction of bioactive compounds. However, this research lines remain only partly connected. Kefir studies are mostly based on fuzzy logic and quality classification; yoghurt studies mainly target pH, acidity, or microbial growth; and chemometric studies on kefir-related beverages generally rely on static calibration models. Therefore, there is still a clear need for a model that treats storage time as an ordered structure and predicts bioactive responses in a functional kefir matrix. The present study addresses this gap by combining alginate-encapsulated black grape seed extract, storage-time-based data organization, chemometric feature transformation, deep temporal learning, and optimizer-guided model tuning for the simultaneous prediction of total phenolic content and antioxidant capacity. This makes the proposed framework different from earlier kefir control models, conventional chemometric calibration studies, and yoghurt-oriented fermentation monitoring approaches.

## 3. Materials and Methods

This section presents the integrated experimental and computational methodology developed to predict phenolic retention and antioxidant capacity in kefir enriched with free and alginate-encapsulated black grape seed extract during refrigerated storage. The experimental phase was structured to generate a controlled repeated-measures dataset from five kefir formulations, three storage intervals, and four independent replicates. The analytical phase included the preparation of kefir samples, alginate encapsulation of black grape seed extract, and measurement of physicochemical, color, textural, and bioactive properties. The computational phase then transformed these measurements into a leakage-safe modelling structure by applying train-only preprocessing, principal component-based chemometric feature extraction, and storage-time sequence construction. Finally, the resulting temporal feature representation was used to train an optimizer-guided deep learning model for the simultaneous prediction of total phenolic content and Trolox-equivalent antioxidant capacity, followed by model comparison, diagnostic evaluation, and validation analyses.

### 3.1. Materials, Grape Seed Extract Preparation, and Alginate Encapsulation

Ultra-high-temperature (UHT) whole cow’s milk was obtained from a commercial dairy company in Izmir, Turkey, and used as the fermented dairy matrix. Food-grade powdered black grape seeds (*Vitis vinifera* L.) were supplied by a commercial producer in Istanbul, Turkey. A freeze-dried kefir starter culture, produced commercially in Poland, was used for fermentation. ABTS (2,2′-azinobis-(3-ethylbenzothiazoline-6-sulfonic acid) diammonium salt), Trolox (6-hydroxy-2,5,7,8-tetramethylchroman-2-carboxylic acid), and gallic acid were obtained from Acros Organics (Morris Plains, NJ, USA). The Folin–Ciocalteu reagent was supplied by Merck (Darmstadt, Germany), and the following chemicals were purchased from Sigma-Aldrich (St. Louis, MO, USA): potassium persulfate ($K_{2}S_{2}O_{8}$), sodium carbonate (${Na}_{2}{CO}_{3}$), sodium alginate, sodium chloride (NaCl), and calcium chloride (${CaCl}_{2}$). Unless otherwise stated, all chemicals and reagents used throughout the study were of analytical grade.

The aqueous black grape seed extract was prepared by suspending 3 g of black grape seed powder in 210 mL of distilled water at a ratio of 1:70 (*w*/*v*). This was stirred continuously at 400 rpm for 60 min at room temperature (25 ± 1 °C) to encourage the release of water-soluble phenolic compounds. After extraction, the mixture was left to settle, then clarified in two steps: first, coarse filtration to remove large particles, followed by filtration through Whatman No. 1 filter paper. The freshly prepared extract was then used immediately for alginate encapsulation.

**Figure 1:**
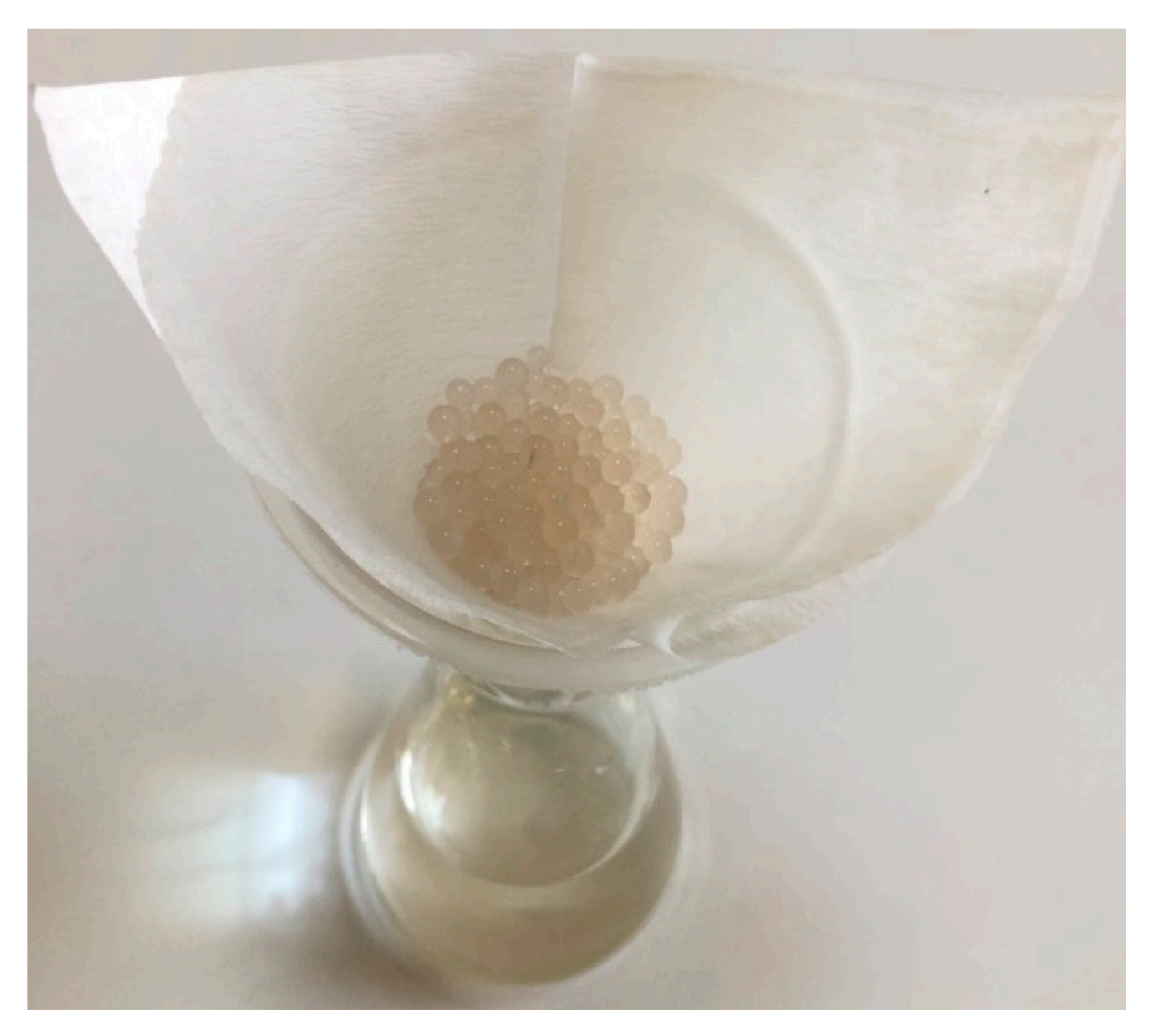
Alginate beads loaded with black grape seed extract.

Alginate encapsulation was performed by ionic gelation with slight modifications to previously reported procedures for bioactive-loaded food hydrogels [31]. Sodium al-ginate (2 g) and NaCl (1 g) were dissolved in 100 mL of freshly prepared black grape seed extract under constant stirring. The mixture was maintained at 50 °C for 3 min and homogenized for 2 min using a laboratory homogenizer (Homogenizer T 65, IKA, Staufen, Germany) to obtain a uniform polymer-extract phase. The alginate-extract solution was transferred into a 21 G syringe and dropped into a chilled 2% (*w*/*v*) ${CaCl}_{2}$ solution under continuous agitation at 200 rpm. The syringe tip was kept approximately 10 cm above the calcium chloride solution. The formed beads were kept in the cross-linking solution for 30 min to complete gelation, separated by filtration, gently washed with distilled water to remove residual calcium ions and loosely bound extract, and incorporated into kefir formulations without a drying step. The visual appearance of the alginate beads loaded with black grape seed extract is shown in Figure 1.

### 3.2. Bead Characterization and Encapsulation Performance

The retention of phenolic compounds within the alginate beads was quantified through a phenolic mass-balance approach, in which encapsulation efficiency (EE) and loading capacity (LC) were derived from total phenolic content (TPC) measurements of the extract and of the bead-derived fractions. In every case, TPC was determined by the Folin–Ciocalteu assay using gallic acid as the calibration standard, and the phenolic concentration of the extract employed for bead preparation was expressed as milligrams of gallic acid equivalents per milliliter (mg GAE/mL) [32].

The surface phenolic fraction (SP) accounts for the phenolics adhering loosely to the outer bead surface rather than those genuinely entrapped within the gel network. To recover this fraction, 2 g of freshly prepared beads were gently washed with 10 mL of distilled water for 1 min at room temperature; the washing solution was then collected and analyzed for TPC. Total phenolics (TP) were determined on a separate 2 g portion of beads, which was dissolved completely in 2% (*w*/*v*) sodium citrate under mild stirring for 10 min. Citrate sequesters the calcium ions that cross-link the alginate chains, disrupting the calcium–alginate network and liberating the entrapped phenolics for quantification, in line with the extraction principle reported by Flamminii et al. [33]. The resulting solution was filtered and analyzed by the same TPC procedure applied to the surface fraction, so that TP and SP were strictly comparable. Encapsulation efficiency was then obtained from the difference between the two fractions, following the calculation adopted by González Morales et al. [34], as given in

(1) $$EE\;\left(\%\right)=\frac{TP-SP}{TP}\times 100$$

where TP is the total phenolic content released from the dissolved beads and SP is the phenolic content detected in the washing solution. All measurements were carried out in duplicate for each of three independent production batches.

Loading capacity, which relates the amount of entrapped phenolics to the mass of carrier material, was estimated from the same TPC data [32,35]. The quantity of encapsulated phenolics was again taken as the difference between TP and SP, while the dry mass of the beads was recorded after drying at 40 °C until a constant weight was reached. LC was computed according to

(2) $$LC\;\left(\%\right)=\frac{TP-SP}{W}\times 100$$

where TP (mg GAE) represents the total phenolic content of the dissolved beads, SP (mg GAE) represents the surface phenolic content, and W (g) is the dry weight of the beads used for the analysis. All LC determinations for EE were performed in duplicate for each of three independent experimental batches to provide a consistent basis for evaluating the reproducibility of the encapsulation process.

Bead dimensions were characterized on freshly prepared, non-dried beads to reflect the physical state in which they were subsequently added to the kefir. For each batch, ten beads were selected at random, and their diameters were measured with a 150 mm/6″ digital vernier caliper (ElectroPeak, Friendswood, TX, USA); the values were summarized as the mean diameter with its standard deviation (SD) [36]. Because the wet beads were incorporated into the kefir formulations without a drying step, these wet-state diameters correspond directly to the particles present in the final products.

The external morphology and structural integrity of the encapsulated black grape seed (*Vitis vinifera* L.) extract microcapsules were examined by scanning electron microscopy (Quanta FEG 450 field-emission scanning electron microscopy (SEM), Thermo Fisher Scientific, Hillsboro, OR, USA), following the microcapsule imaging protocol described by Yourdkhani et al. [37]. Prior to observation, the microcapsules were dried in a laboratory oven at 40 °C for 24 h and sputter-coated with an approximately 10 nm gold layer to enhance surface conductivity during imaging. Micrographs were acquired under high-vacuum conditions at an accelerating voltage of 10 kV using a backscattered-electron (BSE) detector, with the working distance held between 11.7 and 12.2 mm and magnifications ranging from ×50 to ×500. The resulting images were used to assess particle shape, surface continuity, and the overall structural integrity of the beads.

### 3.3. Kefir Production, Formulation Codes, and Storage Design

Kefir was produced by adapting the freeze-dried starter protocol of Mudoor Sooresh et al. [38], with only minor changes introduced to accommodate the enriched formulations. Activation of the culture came first: the freeze-dried starter was grown in skimmed milk held at 25 °C until the pH fell to 4.6, a step that took roughly 16–18 h, after which the activated culture was refrigerated at 4 °C overnight. For the milk base, UHT whole milk was heat-treated at 85 °C for 10 min and then cooled to the inoculation temperature of 25 °C. Once cooled, the milk received either free black grape seed extract or alginate-encapsulated black grape seed microspheres at one of two levels, 1% or 3% (*w*/*v*), and each batch was stirred until the added material was evenly dispersed through the matrix. The enriched milk was inoculated with activated kefir culture at 3% (*v*/*v*), portioned into sterile 500 mL glass jars, and fermented at 25 °C until the pH again reached 4.6. Fermented samples were moved without delay to 4 °C; the first day after cooling was taken as day 1 of storage, and physicochemical characteristics, antioxidant activity, and textural properties were measured on storage days 1, 7, and 14.

**Figure 2:**
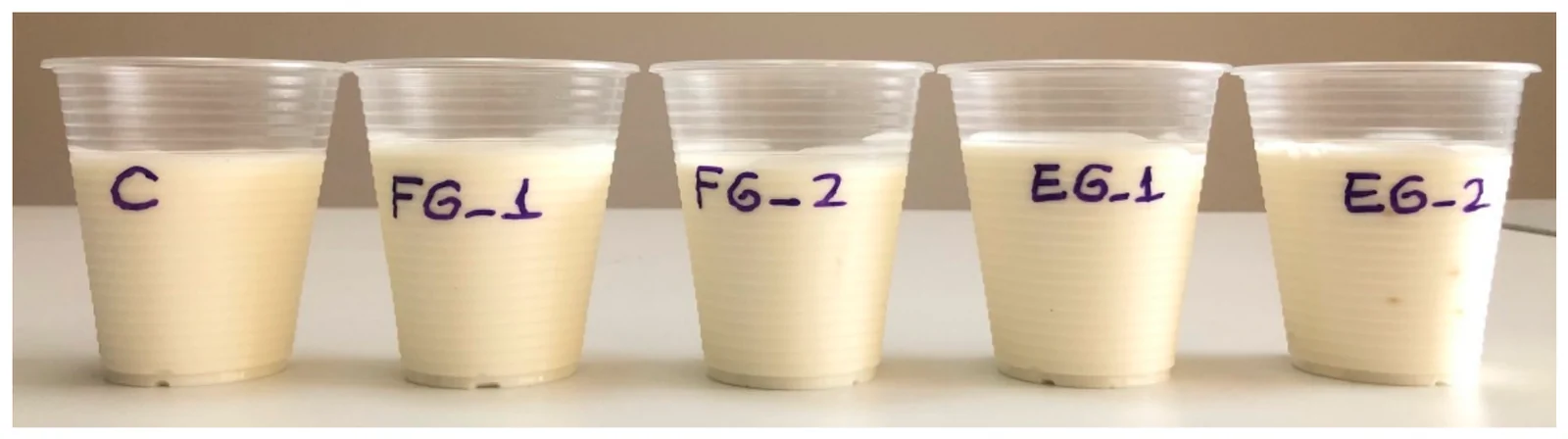
Appearance of the five experimental kefir formulations after manufacture: C, control kefir without added extract; FG-1 and FG-2, kefir enriched with 1% and 3% (*w*/*v*) free black grape seed extract, respectively; and EG-1 and EG-2, kefir enriched with 1% and 3% (*w*/*v*) alginate-encapsulated black grape seed extract, respectively.

As shown in Table 1, the five formulations were designed to represent both the type and dose of black grape seed extract: an unsupplemented control (C), free extract at 1% and 3% (FG-1 and FG-2), and encapsulated extract at the same two levels (EG-1 and EG-2). The appearance of the freshly manufactured kefir samples is presented in Figure 2.

Crossing these five groups with the three storage days and four independent replicates per group-day combination fixed the size of the dataset. The total number of analytical observations was therefore

(3) $$N=n_{g}\;n_{t}\;n_{r}=5\times 3\times 4=60,$$

where $n_{g}$, $n_{t}$, and $n_{r}$ denote the numbers of formulation groups, storage days, and replicates, respectively. This set of 60 observations underpinned both the descriptive statistical analyses and the construction of the predictive model. The four replicates at each formulation–day combination were independent fermentation batches rather than repeated measurements of a single batch, so that the reported variability reflects biological and processing variability. The 1% and 3% (*w*/*v*) extract levels were chosen to bracket a low and a moderate functional dose without perturbing the fermentation endpoint, and the 14-day window sampled on Days 1, 7 and 14 spans the typical refrigerated shelf life of fresh kefir while capturing its early, mid and late storage phases. To keep each formulation traceable to its corresponding model input, the labels C, FG-1, FG-2, EG-1, and EG-2 were carried unchanged through the experimental, statistical, and computational stages of the study. The complete dataset used for the descriptive and predictive analyses is provided as Dataset_S1 in the Supplementary Materials.

### 3.4. Physicochemical, Color, Textural, and Bioactive Measurements

Every sample was characterized through the same panel of analyses at each storage interval, and the resulting variables were assigned two distinct roles in the study. Ten descriptors served as model inputs, pH, water-holding capacity (WHC), titratable acidity (TA), firmness, consistency, cohesiveness, work of cohesion, and the CIELab coordinates $L^{*}$, $a^{*}$, and $b^{*}$, so that the input vector of observation i was

(4) $$x_{i}=[\;pH,\;WHC,\;TA,\;\mathrm{firmness},\;\mathrm{consistency},\;\mathrm{cohesiveness},\;\mathrm{work}\;\mathrm{of}\;\mathrm{cohesion},\;L^{*},\;a^{*},\;b^{*}\;{]}^{⊤}\in R^{10}$$

The two bioactive responses, total phenolic content (TPC, mg GAE/L) and Trolox-equivalent antioxidant capacity (TEAC, expressed as mM), formed the supervised target vector

(5) $$y_{i}=[\;y_{i}^{TPC},\;y_{i}^{TEAC}\;{]}^{⊤}\in R^{2}$$

The total color difference ($\Delta E_{ab}^{*}$) was treated separately: it was retained for the descriptive interpretation of storage stability but deliberately excluded from the predictor set, because its pronounced within-group variability would have introduced noise into the multi-output learning stage rather than a useful signal.

Titratable acidity was determined by the procedure of Case et al. [39] and expressed as lactic acid equivalents, while pH was measured potentiometrically with a digital pH meter (Lab 860, Schott Instruments, Mainz, Germany). Water-holding capacity was assessed following Bielska et al. [40]: 10 g of each sample was centrifuged at 4100× *g* for 30 min at 4 °C, after which the separated whey was carefully decanted and weighed. WHC was obtained from

(6) $$WHC\;\left(\%\right)=\left(1-\frac{W_{whey}}{W_{0}}\right)\times 100$$

where $W_{whey}$ (g) is the mass of whey released during centrifugation and $W_{0}$ (g) is the mass of the kefir sample before centrifugation. Higher values therefore indicate a gel network that retains serum more effectively.

Surface color was followed across refrigerated storage on days 1, 7, and 14 using a spectrophotometric colorimeter (Model CS-410, CHN Spec, Hangzhou, China) operating in the CIELab system under D65 illumination with a 10° standard observer. The recorded coordinates were $L^{*}$ (lightness), $a^{*}$ (redness-greenness), and $b^{*}$ (yellowness-blueness) [41]. Overall color change relative to the initial state was quantified as the Euclidean distance between the color vector at storage day t and the day-1 reference vector, following De Flaviis and Sacchetti [42], as given in

(7) $$\Delta E_{ab}^{*}=\sqrt{(L_{t}^{*}-L_{1}^{*}{)}^{2}+(a_{t}^{*}-a_{1}^{*}{)}^{2}+(b_{t}^{*}-b_{1}^{*}{)}^{2}}$$

where ($L_{1}^{*}$, $a_{1}^{*}$, $b_{1}^{*}$) denotes the color coordinates measured on day 1 and ($L_{t}^{*}$, $a_{t}^{*}$, $b_{t}^{*}$) those measured on storage day t=1, 7, or 14. Color readings were taken in triplicate for every formulation at each storage interval.

Turning to texture, firmness, consistency, cohesiveness, and work of cohesion were measured with a texture analyzer (TA-XT plusC, Stable Micro Systems, Godalming, Surrey, UK) equipped with a 5 kg load cell, using a protocol modified from Glibowski and Kowalska [43]. For each determination, 20 mL of kefir was transferred into a plastic container (45 mm diameter × 25.5 mm height), giving a sample height of approximately 18 mm. Measurements were performed at 25 °C with a 35 mm diameter cylindrical probe, which compressed the sample to a depth of 10 mm at a test speed of 1.0 mm$s^{-1}$. Texture profile analysis was carried out in back-extrusion mode, and the force–time curves were processed with texture exponent software (version 8.1.9.0, Stable Micro Systems). Two independent samples were prepared per formulation, each subjected to four instrumental measurements, and the results are reported as mean values.

The first of the two bioactive responses, antioxidant capacity, was determined by the ABTS Trolox-equivalent antioxidant capacity (TEAC) assay. The ABTS^+^ radical cation was generated by reacting to a 7 mM ABTS solution with 2.45 mM potassium persulfate and allowing the mixture to react under appropriate conditions. Before analysis, the radical solution was diluted with phosphate-buffered saline (PBS, pH 7.4) until its absorbance at 734 nm reached 0.70 ± 0.02, and it was equilibrated at 30 °C. Antioxidant activity was then evaluated from the extent to which the sample antioxidants decolorized the ABTS^+^ radical, with absorbance recorded at 734 nm on a ultraviolet–visible (UV–Vis) spectrophotometer (Shimadzu Scientific Instruments, Tokyo, Japan). Values were derived from a Trolox (6-hydroxy-2,5,7,8-tetramethylchroman-2-carboxylic acid) calibration curve and expressed as mmol Trolox equivalents (TE) per liter, numerically identical to µmol TE/mL, in accordance with Re et al. [44]. For consistency, antioxidant capacity is reported throughout this work as TEAC (mM), where 1 mM TE corresponds to 1 mmol Trolox equivalents per liter; the values therefore express the radical-scavenging capacity of each sample as the concentration of Trolox that would produce an equivalent decolorization of the ABTS^+^ radical.

The second response, total phenolic content, was quantified by the Folin–Ciocalteu assay following Singleton and Rossi [32], using gallic acid as the reference standard for calibration. Once the reaction was complete, absorbance was read at 760 nm on a Shimadzu UV–Vis spectrophotometer (Shimadzu Scientific Instruments, Inc., Tokyo, Japan). Concentrations were interpolated from the gallic acid standard curve and reported as milligrams of gallic acid equivalents per liter of sample (mg GAE/L). Applying this panel of analyses to the five formulations across three storage days with four independent replicates produced the complete input–response dataset of 60 paired input–response records that was subsequently carried into the chemometric and deep learning stages.

### 3.5. Computational Framework: Dataset Construction, Leakage-Safe Preprocessing and Exploratory Analysis

The computational stage maps the analytical measurements onto the two bioactive endpoints through an ordered composition of operators; each fitted on the training partition alone or fixed a priori:

(8) $$\hat{y}=\left(\Phi_{\Theta^{⋆}}\circ T_{seq}\circ \Pi_{PCA}\circ S_{std}\right)\left(X\right),\;\;\Theta^{⋆}=arg\underset{\theta \in \Omega }{min}J\left(\theta \right)$$

Standardization ($S_{std}$) is followed by chemometric orthogonalisation ($\Pi_{PCA}$), storage-time sequence encoding ($T_{seq}$), and a convolutional–recurrent network Φ whose hyperparameters are selected by minimizing the cross-validated loss J. Each operator is developed below.

Every observation carries its experimental provenance, giving the dataset

(9) $$D=\{\left(x_{i},y_{i},g_{i},t_{i},r_{i}\right){\}}_{i=1}^{N},\;\;x_{i}\in R^{p},\;\;y_{i}\in R^{2},\;\;N=60,\;\;p=10,$$

with group $g_{i}$, storage day $t_{i}=1,\;7,\;14$ and replicate $r_{i}=1,\ldots ,4$. Retaining this triplet is what later permits an unambiguous reshaping into temporal sequences and prevents any replicate from being split across partitions.

Missing entries in descriptive variables were imputed by the group-wise mean, preserving the between-formulation contrast the model must learn:

(10) $${\tilde{x}}_{ij}=\frac{1}{\left|G_{j}\left(g_{i}\right)\right|}\sum_{m\in G_{j}\left(g_{i}\right)}x_{mj}$$

Because the descriptors span several orders of magnitude, all inputs were z-score standardized using location and scale statistics estimated *exclusively* on the training partition:

(11) $$x_{ij}^{*}=\frac{x_{ij}-\mu_{j}}{s_{j}},\;\;\mu_{j}=\frac{1}{N_{train}}\;\;\sum_{i\in D_{train}}\;\;x_{ij},\;\;s_{j}=\sqrt{\frac{1}{N_{train}-1}\;\;\sum_{i\in D_{train}}\;\;(x_{ij}-\mu_{j}{)}^{2}}$$

The same train-only rule governed principal component analysis (PCA) loadings, target scaling and augmentation statistics, so that no quantity derived from a held-out sample could reach the training stage, a discipline that is decisive in small food datasets, where even a mild leak inflates apparent accuracy and yields error estimates that fail to reproduce [45,46].

Three exploratory analyses characterized the data before modelling. Replicate dispersion within each formulation, day cell was quantified by the coefficient of variation, which doubles as a precision measure of the analytical protocol and a robustness measure of the formulation:

(12) $$CV_{g,t,j}\;\left(\%\right)=100\times \frac{s_{g,t,j}}{{\bar{x}}_{g,t,j}}.$$

Descriptor–response association was screened by the Pearson coefficient for linear covariation and by the Spearman rank coefficient for monotonic dependence,

(13) $$r_{uv}=\frac{\sum_{i}\left(u_{i}-\bar{u}\right)\left(v_{i}-\bar{v}\right)}{\sqrt{\sum_{i}{\left(u_{i}-\bar{u}\right)}^{2}}\sqrt{\sum_{i}{\left(v_{i}-\bar{v}\right)}^{2}}},\;\;\rho_{uv}=1-\frac{6\sum_{i=1}^{N}d_{i}^{2}}{N\left(N^{2}-1\right)},$$

the latter being the more informative of the two here, since phenolic degradation is curvilinear and a descriptor may track the targets faithfully in rank order while showing only a modest linear coefficient. Formulation effects were tested by one-way analysis of variance (ANOVA) with an accompanying effect size,

(14) $$F=\frac{SS_{B}/\left(k-1\right)}{SS_{W}/\left(N-k\right)},\;\;\eta^{2}=\frac{SS_{B}}{SS_{T}}=\frac{\sum_{g=1}^{k}n_{g}{\left({\bar{x}}_{g}-\bar{x}\right)}^{2}}{\sum_{i=1}^{N}{\left(x_{i}-\bar{x}\right)}^{2}},$$

with k=5 groups and pairwise contrasts protected by Bonferroni correction. With only four replicates per cell, the eta-squared statistic is the more trustworthy guide: it reports the share of variance a descriptor explains, and therefore whether it carries enough formulation-specific signal to be worth passing to the network. The exploratory stage was completed by group-wise boxplots, day-wise violin plots, pairwise scatter density plots and mean value heatmaps, which together expose distributional shape, dispersion and outliers before any model is fitted.

### 3.6. Chemometric Feature Extraction by Principal Component Analysis

The descriptor panel is redundant by construction, as firmness, consistency, cohesiveness and work of cohesion all probe the same casein gel network, which inflates the condition number of the input Gram matrix and destabilizes gradient-based training. PCA was therefore interposed between measurement and learning as a numerical conditioning step rather than a cosmetic reduction [19]. From the standardized matrix, the covariance matrix and its spectral decomposition are

(15) $$\hat{\Sigma }=\frac{1}{N-1}{\left(X^{*}\right)}^{⊤}X^{*}\in R^{p\times p},\;\;\hat{\Sigma }W=W\Lambda ,\;\;\Lambda =diag\left(\lambda_{1},\ldots ,\lambda_{p}\right),\;\;\lambda_{1}\geq \cdots \geq \lambda_{p}\geq 0$$

The variance carried by each component, and the criterion fixing how many to retain, are

(16) $$EVR_{j}=\frac{\lambda_{j}}{\sum_{m=1}^{p}\lambda_{m}},\;\;q=min\left\{k:\sum_{j=1}^{k}EVR_{j}\geq 0.85\right\}$$

which was satisfied at q=5, retaining approximately 86% of the total variance while halving the input dimension. Projection gives the score matrix

(17) $$Z=X^{*}W_{q}\in R^{N\times q},\;\;q=5$$

whose columns are mutually uncorrelated by construction. The loadings remain physically readable, the leading component is dominated by the texture block, the second by moisture retention and acidity, so the network operates on latent variables that retain food science meaning rather than on an opaque embedding. Scree, cumulative variance and biplot representations were retained as chemometric evidence for the choice of q.

### 3.7. Storage-Time Encoding and Dual-Target PCA–CNN–LSTM Prediction Architecture

Shelf-life prediction was framed as a short-sequence problem rather than a table of independent rows: a Day-14 sample is the endpoint of a trajectory, and the drift of its texture, acidity and color between Days 1 and 7 carries information about how much phenolic material survived. For each group and replicate the PCA scores were ordered by storage day and concatenated with the bioactive history, with the Day-14 targets masked out of the input to preclude leakage:

(18) $${\tilde{b}}_{g,r,t}=\{\begin{array}{ll} b_{g,r,t}, & t\in \{1,7\},\\ 0_{2}, & t=14, \end{array}\;\;S_{g,r}=\left[\begin{array}{cc} z_{g,r,1}^{⊤} & {\tilde{b}}_{g,r,1}^{⊤}\\ z_{g,r,7}^{⊤} & {\tilde{b}}_{g,r,7}^{⊤}\\ z_{g,r,14}^{⊤} & {\tilde{b}}_{g,r,14}^{⊤} \end{array}\right]\in R^{3\times \left(q+2\right)},\;\;y_{g,r}=b_{g,r,14}\in R^{2}$$

Storage day thus indexes the temporal axis and the chemometric scores plus bioactive history the feature axis, yielding 20 base sequences. The masking is not a technicality: it guarantees that reported accuracy reflects prediction rather than the model reading its own answer from the input.

With four replicates per group, a random split can strand all extreme values on one side and force extrapolation. A median-anchored rule therefore assigned to the test set the replicate whose Day-14 TPC lies closest to its group median,

(19) $$r_{g}^{test}=arg\underset{r\in \{1,2,3,4\}}{min}|TPC_{g,r,14}-{median}_{r}\left(TPC_{g,r,14}\right)|$$

giving 15 training and 5 test sequences, one representative per formulation, with every test target inside the interpolation range of the training data. Because each replicate’s three storage days are assembled into a single sequence, a replicate is assigned in full to either the training or the test partition and is never split across the two. The training partition was expanded four-fold by controlled perturbation, additive Gaussian noise on the inputs and multiplicative jitter on the targets representing instrument precision and replicate variability, respectively [47]:

(20) $${\tilde{S}}_{i,k}=S_{i}+E_{i,k},\;\;E_{i,k}∼N\left(0,\sigma_{\epsilon }^{2}I\right);\;\;{\tilde{y}}_{i,k}=y_{i}⊙u_{i,k},\;\;u_{i,k}∼U\left(1-\delta ,1+\delta \right)$$

with noise amplitude 0.02, jitter 0.03 and k=1, …, 4, giving 60 training sequences. All augmentation statistics were estimated on the training partition alone; the Day-14 bioactive entries remained masked throughout, so that the perturbation never touched the quantity being predicted; and the five held-out test sequences were left entirely unaltered. Targets were finally mapped to the unit interval by training set extrema,

(21) $$y_{ij}^{sc}=\frac{y_{ij}-y_{j,min}^{train}}{y_{j,max}^{train}-y_{j,min}^{train}}$$

placing TPC (order $10^{3}$ mg GAE/L) and TEAC (order $10^{1}$ mM) on a common footing so that neither dominates the shared loss; all predictions were inverse-transformed to original units before any metric was computed.

The predictor consumes this tensor and returns both endpoints jointly. A one-dimensional convolution with same-padding first extracts local storage transitions, the Day-1-to-Day-7 and Day-7-to-Day-14 drifts, as f feature maps, followed by batch normalization (BN) [48]:

(22) $$h_{t,l}^{\left(1\right)}=\phi \;\left(\sum_{k=0}^{K-1}\sum_{j=1}^{d}w_{l,k,j}^{\left(1\right)}S_{t+k,j}+b_{l}^{\left(1\right)}\right),\;\;\phi \left(a\right)=max\left(0,a\right);\;\;BN\left(a\right)=\gamma \frac{a-\mu_{B}}{\sqrt{\sigma_{B}^{2}+\epsilon }}+\beta .$$

A long short-term memory (LSTM) layer with *h* gated units then traverses the sequence, its gates deciding what to forget, write and expose at each storage step [18]:

(23) $$f_{t}=\sigma \left(W_{f}\left[h_{t-1},x_{t}\right]+b_{f}\right)$$

(24) $$i_{t}=\sigma \left(W_{i}\left[h_{t-1},x_{t}\right]+b_{i}\right)$$

(25) $${\tilde{c}}_{t}=tanh\left(W_{c}\left[h_{t-1},x_{t}\right]+b_{c}\right)$$

(26) $$c_{t}=f_{t}⊙c_{t-1}+i_{t}⊙{\tilde{c}}_{t}$$

(27) $$o_{t}=\sigma \left(W_{o}\left[h_{t-1},x_{t}\right]+b_{o}\right)$$

(28) $$h_{t}=o_{t}⊙tanh\left(c_{t}\right)$$

The final hidden state condenses the storage trajectory and is passed through a dense layer with dropout to a two-neuron linear head,

(29) $${\hat{y}}^{sc}=W_{out}\;\phi \left(W_{d}h_{T}+b_{d}\right)+b_{out}\in R^{2},\;\;L\left(\Theta \right)=\frac{1}{n}\sum_{i=1}^{n}\sum_{j=1}^{2}({\hat{y}}_{ij}^{sc}\left(\Theta \right)-y_{ij}^{sc}{)}^{2}$$

Predicting both responses from one shared representation is deliberate: phenolics are the dominant radical-scavenging species in this matrix, so TPC and TEAC are chemically coupled and each target regularizes the other. Equal loss weights are justified because both were mapped to a common range.

### 3.8. Crested Porcupine Optimizer (CPO) for Evolutionary Hyperparameter Tuning: Framework Synthesis and Validation Pipelines

Six hyperparameters govern the behavior of the architecture and were encoded as a candidate solution $\theta ={\left[f,K,h,d,\eta ,B\right]}^{⊤}\in \Omega$ over the bounded search space of Table 2. The bounds in Table 2 follow the structure of the problem: the kernel size is limited to two or three because the sequence has only three time points; the filter and LSTM unit ranges span minimal to generous capacity for 60 training sequences; the learning rate is searched log-uniformly over the range in which Adam trains stably at this scale; and the batch size is bounded above by the training set size. Their joint optimization is a non-convex, mixed integer–continuous problem in which each evaluation costs a full network training, so the fitness was the three-fold cross-validated mean squared error on the training partition,

(30) $$J\left(\theta \right)=\frac{1}{K_{cv}}\sum_{k=1}^{K_{cv}}\frac{1}{n_{k}}\sum_{i\in V_{k}}∥{\hat{y}}_{i}^{sc}\left(\theta \right)-y_{i}^{sc}{∥}_{2}^{2}\;,\;\;\;K_{cv}=3$$

where $K_{cv}=3$ is the number of cross-validation folds, $V_{k}$ denotes the k-th validation fold, $n_{k}=\left|V_{k}\right|$ its size, and ${\hat{y}}_{i}^{sc}$ and $y_{i}^{sc}$ are the predicted and reference targets in the scaled representation. The identical definition imposed on the competing optimizer so that the comparison measures search behavior alone. The search was run for 15 iterations.

The CPO explores the domain by imitating the graded defensive repertoire of *Hystrix cristata*, escalating from distant visual deterrence to close-range attack [49]. CPO was adopted because, in its original benchmarking, it delivered a superior convergence rate to a broad set of optimizers, including particle swarm optimization and several algorithms that themselves outperform it, across the CEC2014, CEC2017 and CEC2020 suites and six engineering problems [49]. Each candidate is updated by the superposition of four operators and projected back onto the feasible domain,

(31) $$\theta_{i}^{t+1}=\Pi_{\Omega }\left(\theta_{i}^{t}+\Psi_{i,t}^{sight}+\Psi_{i,t}^{sound}+\Psi_{i,t}^{odour}+\Psi_{i,t}^{attack}\right),$$

whose contributions shift progressively from exploration to exploitation,

(32) $$\Psi_{i,t}^{sight}=r_{1}\left(\theta_{\alpha }^{t}-\theta_{i}^{t}\right)$$

(33) $$\Psi_{i,t}^{sound}=r_{2}\left(\theta_{best}^{t}-\left|\theta_{i}^{t}\right|\right)cos\left(2\pi r_{3}\right)$$

(34) $$\Psi_{i,t}^{odor}=r_{4}\left(\theta_{best}^{t}-\theta_{i}^{t}\right)e^{-\kappa t/T}$$

(35) $$\Psi_{i,t}^{attack}=r_{5}\;L\left(\beta \right)⊙\left(\theta_{\alpha }^{t}-\theta_{i}^{t}\right)$$

where $r_{1},\ldots ,\;r_{5}\sim U(0,1)$, $\theta_{\alpha }$ is the current best (alpha) agent, κ controls the exploitation rate, T is the total iteration budget, and the Lévy step (L(β)) is:

(36) $$L\left(\beta \right)=\frac{u}{{\left|v\right|}^{1/\beta }}\;\;\;\;\;\;\;\;\;\;\;\;\;\;u\sim N\left(0,{\sigma_{u}}^{2}\right),\;\;\;\;v\sim N\left(0,{\sigma_{v}}^{2}\right),\;$$

with $\sigma_{v}=1$ and

(37) $$\sigma_{u}={\left[\frac{\Gamma (1+\beta )sin(\frac{\pi \beta }{2})}{\Gamma \left(\frac{1+\beta }{2}\right)\beta 2^{(\beta -1)/2}}\right]}^{1/\beta },\;\;\;\;\beta =1.5$$

with coefficients drawn uniformly on the unit interval, an elite agent, the incumbent best solution, and a Lévy-flight step of exponent 1.5 that produces occasional long jumps and so guards against stagnation in a local basin. The exponential decay in the odor term narrows the search radius as the budget is consumed. What sets CPO apart from fixed-population metaheuristics is cyclic population reduction,

(38) $$N_{p}\left(t\right)=max\left(N_{min},\;\left[N_{p,0}\left(1-t/T\right)\right]\right),\;\;\;t=1,\ldots ,T$$

which retires the weakest agents while retaining a minimum population size to preserve search diversity. Here, $N_{p,0}=10$ denotes the initial population size, while $N_{min}$ represents the minimum retained population size. The ($N_{p,0}-N_{p}(t)$) agents with the highest error—that is, the worst-performing agents—are removed so that the surviving population at iteration t equals $N_{p}\left(t\right)$. This adaptive population contraction progressively concentrates the exploration effort on the best-performing regions of the search space. The consequence is particularly important at the present data scale: because every fitness evaluation requires training a network, an optimizer that allocates a gradually shrinking pool of evaluations to increasingly promising regions can extract greater performance improvement per unit of computation. This mechanism contrasts with standard PSO, which retains a fixed population and therefore cannot explicitly reallocate computational resources by adaptively reducing the number of active search agents. For the benchmark, particle swarm optimization was implemented using the standard inertia–cognition–social update rule [50],

(39) $$v_{i}^{t+1}=\omega v_{i}^{t}+c_{1}r_{1}\left(p_{i}^{t}-\theta_{i}^{t}\right)+c_{2}r_{2}\left(g^{t}-\theta_{i}^{t}\right),\;\;\theta_{i}^{t+1}=\Pi_{\Omega }\left(\theta_{i}^{t}+v_{i}^{t+1}\right)$$

The network configured with the winning hyperparameter vector was trained with Adam [51] under early stopping and learning rate reduction on the validation loss, and training stability was monitored through the training and validation loss trajectories across epochs.

### 3.9. Ablation Design, Performance Metrics and Diagnostic Validation

To establish that each component earns its place, five configurations were evaluated under an identical split, scaling protocol, and evaluation loop, as summarized in Table 3. These configurations comprise the recurrent baseline, the addition of the convolutional block, the inclusion of the PCA front-end, and the hyperparameter-optimized variants driven by PSO and CPO. This progression isolates the architectural contributions first and the optimizer contribution last, such that any difference between the final two models can be attributed solely to search behavior.

The design in Table 3 is deliberately cumulative rather than factorial, and the choice is worth justifying. A full factorial ablation over three binary components and two optimizers would require sixteen configurations, most of which would offer limited scientific value, such as a PCA front-end without a network to condition or a tuned optimizer applied to an architecture that would not be practically deployed. The nested design instead follows the construction logic of the framework itself: each row is the row above it plus exactly one addition, so the difference between any two consecutive configurations is a clean estimate of the marginal value of the component introduced at that step, with everything else held constant.

Two boundaries within the table carry the analytical weight. The first is the M3–M4 boundary, which separates the architectural contributions from the optimization contribution: M1 through M3 share the same default hyperparameters and differ only in structure, so any improvement across them is attributable to the model’s inductive bias and not to tuning. The second is the M4–M5 boundary, which is the study’s central comparison. Here, the architecture, training data, data split, scaling protocol, fitness function, and iteration budget are identical, and the hyperparameter search domain defined in Table 2 is also held constant, leaving the search algorithm as the only factor that changes. Because CPO contracts its population while PSO retains a fixed one, CPO in fact consumes fewer total fitness evaluations over the fifteen iterations; this asymmetry favors CPO in computational cost and does not weaken the comparison. Any difference observed between these two rows, therefore, cannot be attributed to model capacity, to data handling, or to a more favorable problem formulation; it isolates search behavior alone. This is a stricter test than the comparisons usually reported when a new metaheuristic is introduced, and it is the reason the ablation is structured this way.

Performance was quantified in the original analytical units, never on the scaled targets, so that the reported errors are directly interpretable by a food technologist:

(40) $$RMSE_{j}=\sqrt{\frac{1}{n}\sum_{i=1}^{n}{\left(y_{ij}-{\hat{y}}_{ij}\right)}^{2}}$$

(41) $$MAE_{j}=\frac{1}{n}\sum_{i=1}^{n}\left|y_{ij}-{\hat{y}}_{ij}\right|$$

(42) $$MAPE_{j}=\frac{100}{n}\sum_{i=1}^{n}\left|\frac{y_{ij}-{\hat{y}}_{ij}}{y_{ij}}\right|$$

(43) $$R_{j}^{2}=1-\frac{\sum_{i=1}^{n}{\left(y_{ij}-{\hat{y}}_{ij}\right)}^{2}}{\sum_{i=1}^{n}{\left(y_{ij}-{\bar{y}}_{j}\right)}^{2}}$$

The four are reported jointly because each is blind to something the others capture: root mean square error (RMSE) penalizes large deviations quadratically and is the right currency for a quality control alarm, mean absolute error (MAE) reports the error a technician would typically meet, mean absolute percentage error (MAPE) renders TPC and TEAC comparable despite their different scales, and the coefficient of determination measures explained variance. The last was interpreted on the final held-out evaluation and not treated as the primary criterion within folds, where its denominator becomes unstable at very small n.

Aggregate metrics can conceal a badly behaved model, so residuals $e_{ij}=y_{ij}-{\hat{y}}_{ij}$ were subjected to diagnosis. Their normality was assessed graphically by quantile–quantile plots and formally by the Shapiro–Wilk statistic [52],

(44) $$W=\frac{(\sum_{i=1}^{n}a_{i}x_{\left(i\right)}{)}^{2}}{\sum_{i=1}^{n}{\left(x_{i}-\bar{x}\right)}^{2}}$$

in which the ordered residuals are weighted by tabulated coefficients. Normality matters here less as a distributional article of faith than as evidence that no structured, systematically signed error remains, which would signal that some deterministic component of the storage kinetics is still unlearned. Method agreement was then examined by the Bland–Altman procedure [53], the standard instrument for asking whether a new technique may substitute for an established assay,

(45) $${\bar{d}}_{j}=\frac{1}{n}\sum_{i=1}^{n}\left(y_{ij}-{\hat{y}}_{ij}\right),\;\;{LoA}_{j}={\bar{d}}_{j}\pm 1.96\;s_{d,j}$$

A mean difference near zero and limits of agreement (LoA) that remain flat across the concentration range are what license the framework to be described as a non-destructive substitute for the reference assays rather than merely as a good fit. All computations were carried out in Python 3.11.15 with NumPy 2.4.4, pandas 3.0.5, SciPy 1.17.1, scikit-learn 1.8.0 [54] and TensorFlow 2.21.0/Keras 3.15.1 [55], with random seeds fixed for every stochastic operation so that the pipeline is reproducible end to end.

## 4. Results and Discussion

This section is organized to follow the logic of the study itself, moving from the material that was engineered to the model that was built on it. It opens with the alginate beads, whose encapsulation efficiency and surface morphology set the physical stage for everything that follows, and then reports how the five formulations behaved during refrigerated storage, where the consequences of encapsulation become visible in the phenolic and antioxidant trajectories. The storage data are then examined at finer resolution, first through their distributional structure and replicate precision, and subsequently through the correlation and principal component analyses that expose the redundancy in the descriptor panel and justify the chemometric transformation placed ahead of the network. Attention then turns to the predictive framework proper, tracing the hyperparameter search, the training behavior, the accuracy achieved on genuinely held-out samples, and the diagnostic tests that establish its reliability, before the closing ablation isolates the contribution of each component and identifies the source of the model’s performance. The experimental and computational threads are treated not as separate reports but as a single argument, in which each analysis motivates the next.

### 4.1. Encapsulation Performance and Bead Morphology

The ionic gelation procedure retained the greater part of the phenolic payload it was given. Across three independent batches, the beads carried 32.8 ± 0.6 mg GAE per gram of dry solid, of which only 4.1 ± 0.2 mg GAE/g remained loosely bound at the surface, giving an encapsulation efficiency of 86.4 ± 0.5% and a loading capacity of 18.7 ± 0.7%. The efficiency sits towards the upper part of the range reported for alginate systems carrying plant phytochemicals, and the composition of the extract offers a plausible explanation. Proanthocyanidins, the dominant phenolic class in black grape seed, present numerous hydroxyl groups capable of hydrogen bonding and other non-covalent association with the guluronic acid blocks of the alginate chain, so the core is in all likelihood not merely occluded within the gel but partially anchored to it. This distinction matters for what follows. A payload held by physical entrapment alone would be expected to leach steadily from the moment the beads enter an aqueous, acidic matrix, whereas one that is partly bound to the polymer network resists that early loss, which is consistent with the retention behavior observed during storage.

Arguably more informative than either mean value is their dispersion. With standard deviations below 1% across independent production runs, the encapsulation step is reproducible enough to be treated as a controlled variable rather than as a source of noise in the storage experiment. The beads measured 2.95 ± 0.15 mm in diameter, within the range typical of extrusion-produced alginate particles containing plant extracts. Across the thirty beads measured (ten per batch, three independent batches), this diameter corresponded to a coefficient of variation of about 5%, indicating a narrow and homogeneous size distribution. Diameter is not a cosmetic descriptor here, because it governs the surface-area-to-volume ratio and therefore the diffusional path length a phenolic molecule must traverse before reaching the surrounding matrix. The narrow spread indicates a structurally uniform population, which is a precondition for the formulation comparison that follows, since a heterogeneous size distribution would translate directly into heterogeneous release kinetics and confound the contrast between encapsulated and free extract.

**Figure 3:**
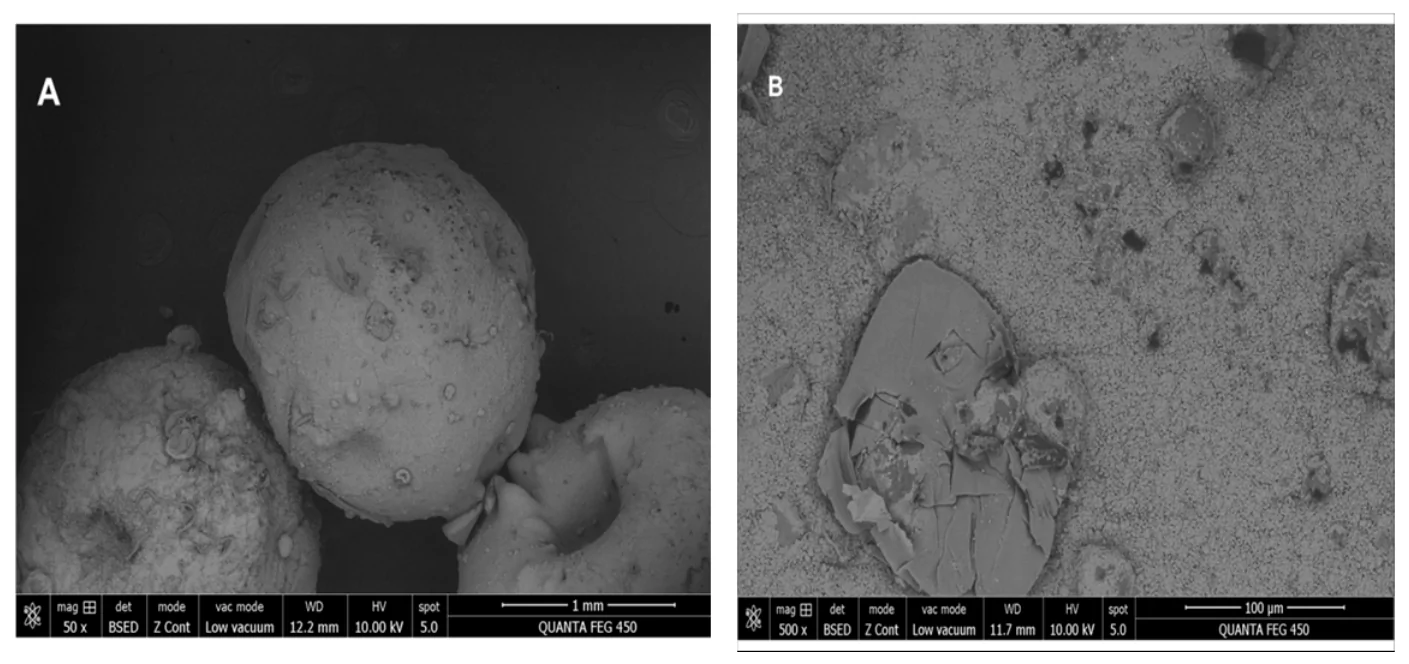
SEM micrographs of dried alginate beads loaded with black grape seed extract at (**A**) ×50 and (**B**) ×500 magnification.

Electron microscopy confirms that the beads survived encapsulation and drying with their structure intact. Figure 3 presents the micrographs at two magnifications. At the lower magnification the beads appear predominantly spherical and comparable in size, with no aggregation and no gross deformation, which corroborates the caliper measurements. The higher the magnification, the more consequential the view. The surface is compact and continuous, with a fine granular texture and no cracks, macropores, or fissures penetrating the wall. Such a morphology is characteristic of calcium alginate formed by ionic gelation, in which the rapid ingress of calcium ions at the droplet interface generates a dense outer skin before the interior has fully gelled. Minor undulations and localized protrusions are visible, and these are most plausibly attributed to dehydration during sample preparation, since hydrogels shrink anisotropically on drying and the imaged surface is not the hydrated one that exists within the product. The absence of continuous porosity is the structurally meaningful observation, because it means the phenolic core is separated from the acidic, oxygenated kefir matrix by an unbroken barrier. This is the physical mechanism to which the retention and stability differences reported below can be traced, and it converts what would otherwise be a purely empirical formulation ranking into one with a structural explanation.

### 4.2. Storage Dynamics of Phenolic Content and Antioxidant Capacity

**Figure 4:**
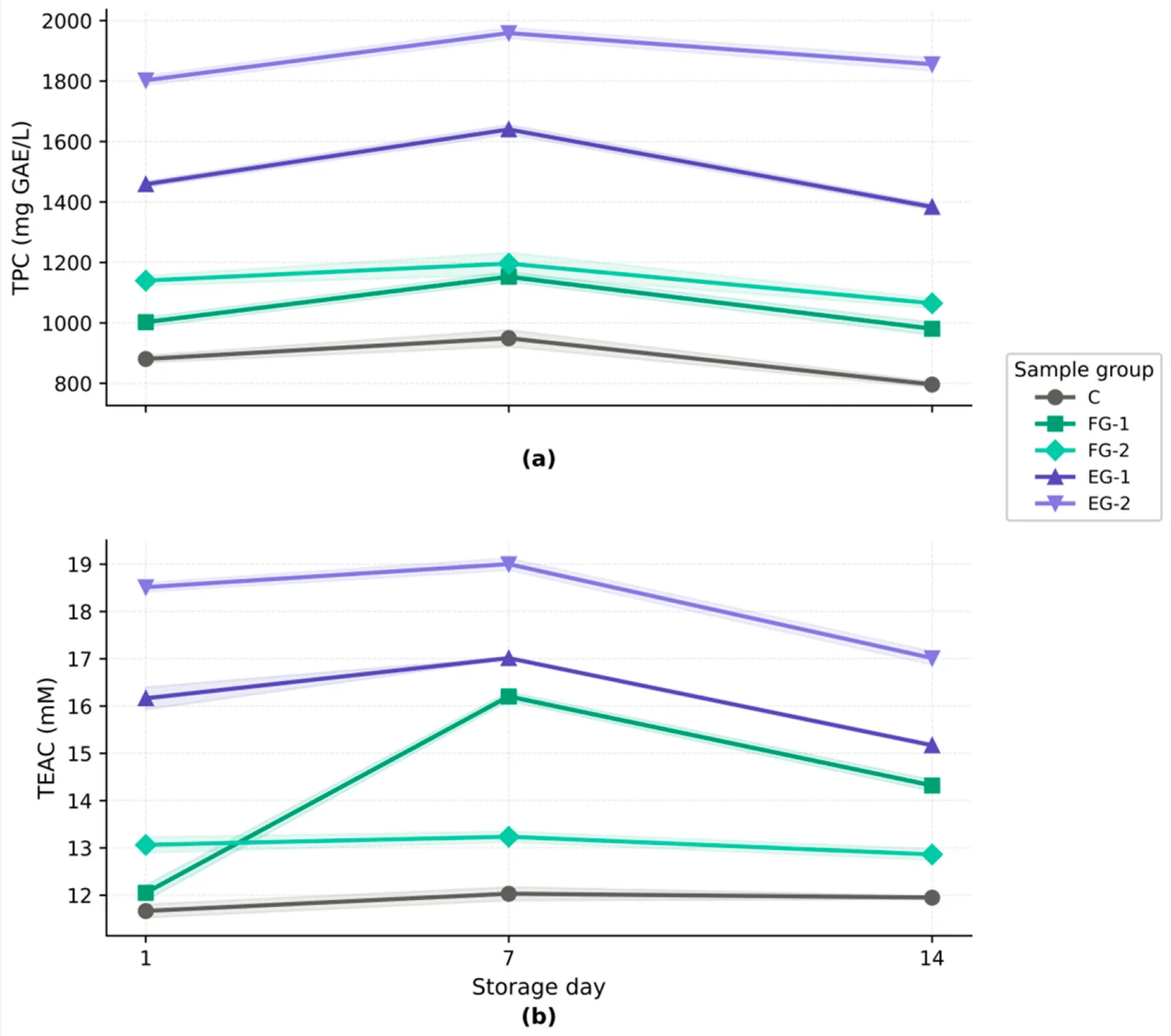
Storage evolution of (**a**) TPC and (**b**) TEAC in the five kefir formulations. Bands denote replicate dispersion.

The barrier inferred from the micrographs manifests directly in the storage data. Figure 4 traces the evolution of both bioactive endpoints over the fourteen-day period and establishes an ordering that persists at every sampling point, with the control lowest, the free-extract formulations intermediate, and the encapsulated formulations highest. This ordering appears to be governed by delivery mode at least as strongly as by dose. EG-2 contained 1802.5 mg GAE/L on Day 1, increased to 1958.8 mg GAE/L on Day 7, and still retained 1855.6 mg GAE/L on Day 14, whereas FG-2, which received the same 3% dose as free extract, never exceeded 1196.2 mg GAE/L. At equal extract loading, encapsulation therefore resulted in approximately 60% more measurable phenolic material in the finished kefir. This difference cannot be attributed to a higher extract dose within the beads, since the loading was matched by design. Rather, free polyphenols introduced into the matrix as solutes are immediately exposed to interactions with milk proteins and oxidative processes, potentially rendering a fraction of the phenolic pool analytically undetectable soon after incorporation. The encapsulated formulations, therefore, preserve not merely phenolic concentration but also the measurable and functionally retained bioactive fraction.

Every enriched formulation traced a non-monotonic path, rising to a Day-7 maximum before declining, and this shape is chemically informative rather than an assay artefact. Continued acidification and proteolysis during the first storage week progressively release phenolics that were initially sequestered by caseins or held within the alginate network, transiently raising the extractable fraction that the Folin–Ciocalteu reagent detects [56]. Thereafter the liberated molecules, now stripped of both the protein complex and the bead wall, undergo oxidation and polymerization, and the measurable pool contracts. The two processes overlap, so the Day-7 peak marks the point at which liberation ceases to outpace degradation rather than any discrete event in the matrix. The magnitude of the subsequent contraction is where the delivery systems separate decisively. Between Day 7 and Day 14 the control lost 16.1% of its phenolic signal and FG-2 lost 11.0%, while EG-2 surrendered only 5.3%. The encapsulated formulations are not simply richer; they decay more slowly, and it is the decay rate rather than the initial load that governs shelf-life performance. The residual signal in the control, between 796 and 949 mg GAE/L, should not be read as endogenous polyphenol, since the Folin reagent responds to milk-derived tyrosine residues and peptides [57]. This reactivity sets a matrix-specific baseline against which every other group must be interpreted, and it also explains why the control declines at all, as the same proteolysis that liberates bound phenolics in the enriched groups alters the peptide pool that the reagent detects in the unenriched one.

**Figure 5:**
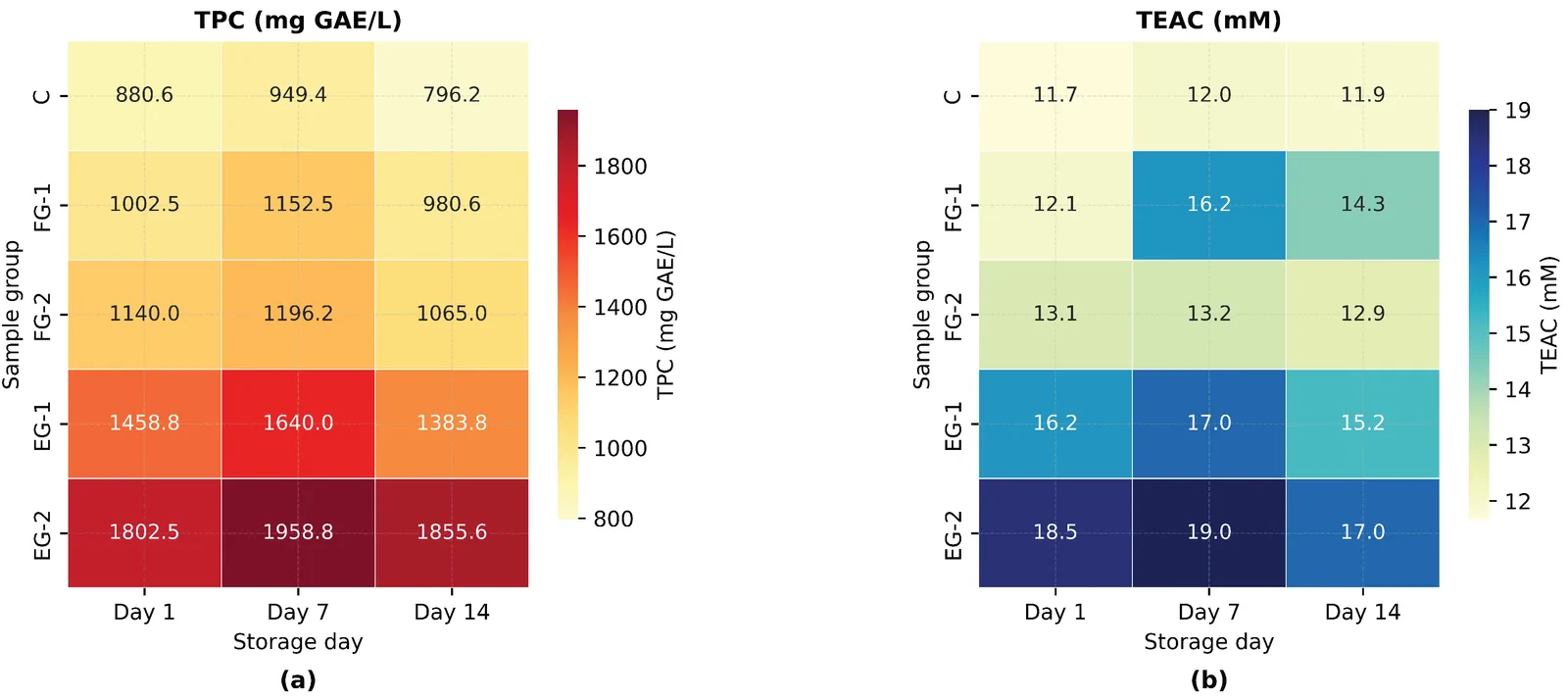
Mean (**a**) TPC and (**b**) TEAC for each formulation and storage day.

Figure 5 resolves the same data into a formulation-by-day grid and makes the consistency of the hierarchy unmistakable. No group changes rank at any time point, which is a stronger statement than the trajectory plot alone conveys. Had encapsulation merely delayed release, one would expect the free-extract formulations to overtake the encapsulated ones at some late point as their beads gradually surrendered a payload that the free groups had already made available; no such crossover occurs. The beads are not slowing the arrival of the phenolics so much as protecting them once they arrive, which is a different and more valuable property in a product that is consumed within its labeled shelf life rather than digested at a controlled rate.

Antioxidant capacity mirrored the phenolic trajectories, as the tight coupling between the two responses would demand, and preserved the same hierarchy. EG-2 peaked at 19.0 mM and retained 17.0 mM at Day 14, while the control remained essentially flat between 11.7 and 12.0 mM. One departure from the pattern is worth dwelling on, because it reveals a mechanism that the phenolic data alone cannot. FG-1 climbed from 12.1 to 16.2 mM between Days 1 and 7, a gain of 34% that far outstrips its concurrent phenolic increase. A disproportionate rise in radical scavenging without a matching rise in total phenolics signals a change in composition rather than in concentration. The most likely mechanism is acid-catalyzed cleavage of high-molecular-weight proanthocyanidins into catechin and epicatechin monomers, which quench the ABTS radical far more efficiently per unit mass than the polymers from which they derive [58]. The Folin assay registers the total phenolic pool with little regard to its degree of polymerization, whereas the ABTS assay is sensitive to the accessibility of the hydroxyl groups performing the scavenging, so a depolymerization event raises the latter while barely disturbing the former. In the free formulations these polymers are exposed directly to the acidifying matrix, whereas the alginate wall moderates that exposure, and this is precisely why EG-1 and EG-2 follow smoother and more proportionate trajectories. The observation carries a practical implication: an antioxidant claim based on TPC alone would misrepresent the functional state of a free-extract product during its first storage week.

### 4.3. Distributional Structure and Analytical Precision

**Figure 6:**
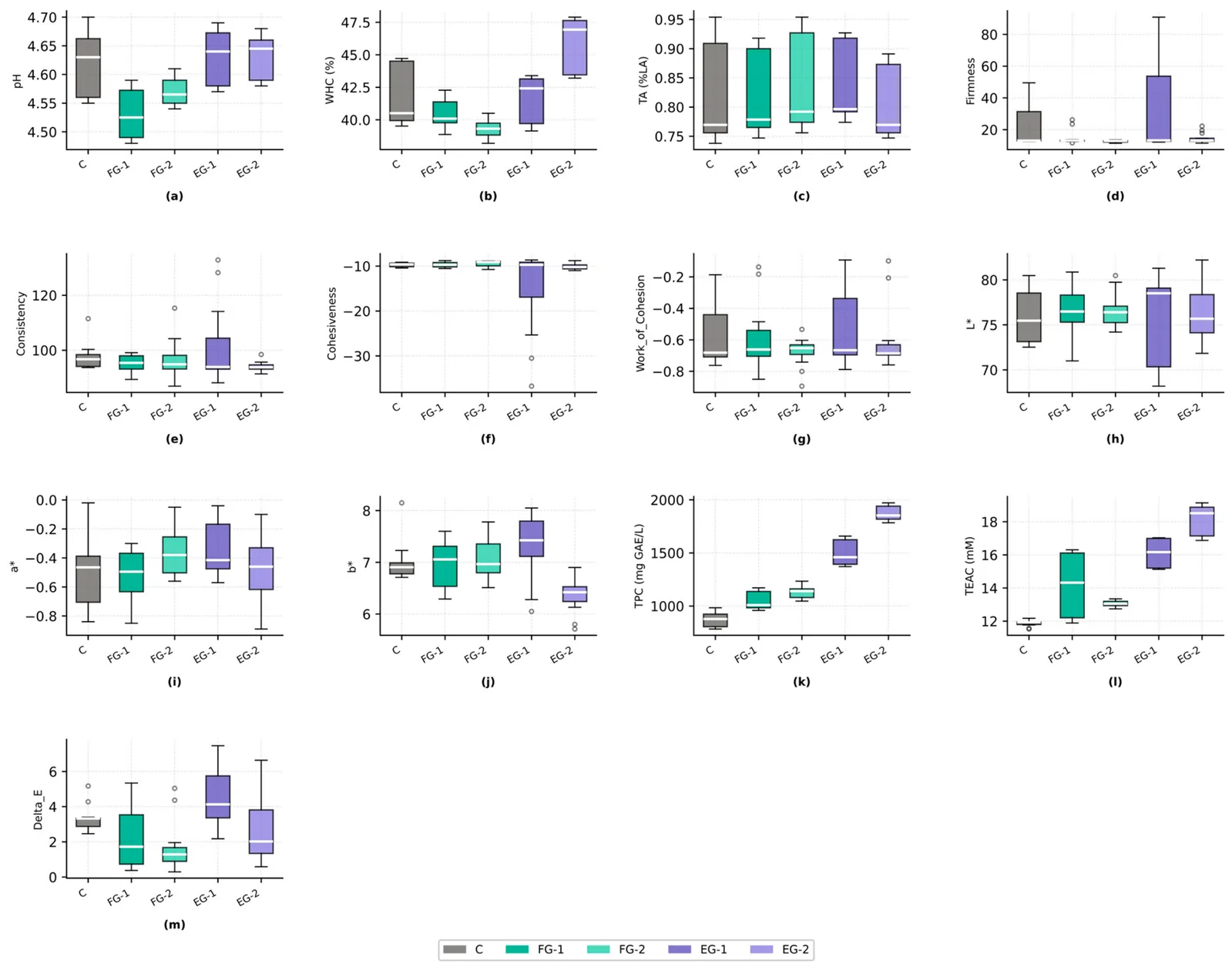
Distribution of the thirteen measured variables across the five formulations. (C, FG-1, FG-2, EG-1, EG-2), shown as box plots: (**a**) pH; (**b**) water-holding capacity, WHC (%); (**c**) titratable acidity, TA (% lactic acid); (**d**) firmness; (**e**) consistency; (**f**) cohesiveness; (**g**) work of cohesion; (**h**) lightness, L*; (**i**) redness–greenness, a*; (**j**) yellowness–blueness, b*; (**k**) total phenolic content, TPC (mg GAE/L); (**l**) Trolox-equivalent antioxidant capacity, TEAC (mM); and (**m**) total colour difference, ΔE*ab. In each box, the central line denotes the median, the box the interquartile range (IQR), the whiskers the values within 1.5 × IQR, and open circles the outliers.

The storage trajectories summarized the formulations through their group means, but a model trained on sixty individual observations is shaped by the full distribution behind those means, and it is worth examining that distribution before turning to the analyses that feed the network. Examined variable by variable in Figure 6, the formulations diverge far more in what they deliver than in what they are. TPC and TEAC separate the five groups with almost no overlap, whereas pH remains confined between 4.48 and 4.70 and titratable acidity between 0.75 and 0.95% across every treatment. Enrichment therefore raised the antioxidant payload substantially without displacing the fermentation endpoint that defines the product. This is not a trivial outcome. Polyphenols are known to interact with lactic acid bacteria, and a sufficiently large addition could in principle inhibit acidification and yield a product that is no longer recognizably kefir; the narrowness of the pH and acidity bands demonstrates that the doses used here remain below that threshold and that the encapsulated formulations in particular deliver their payload without perturbing the fermentation at all.

**Figure 7:**
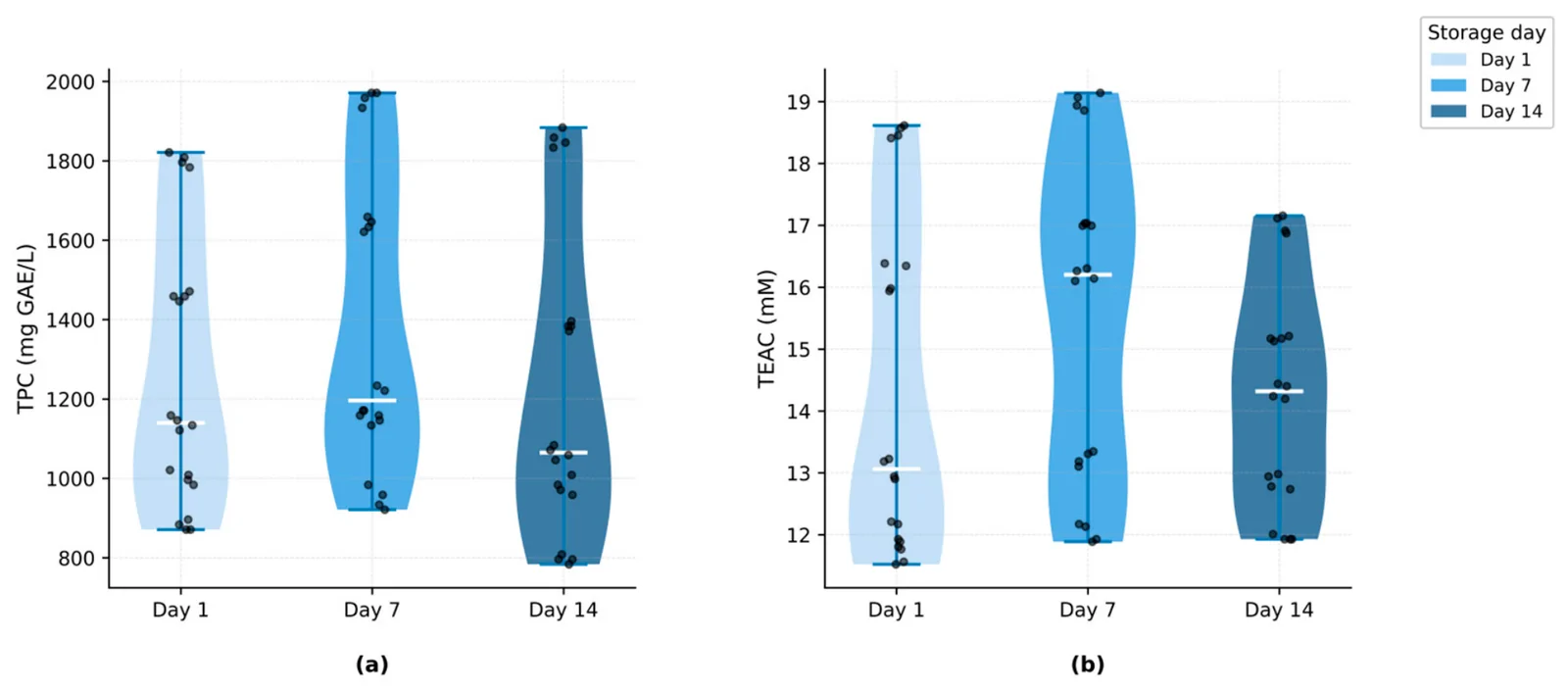
Distribution of (**a**) TPC and (**b**) TEAC by storage day, pooled across formulations.

The textural descriptors behave in a manner that anticipates their later modeling difficulties. Firmness and consistency are strongly right-skewed, most conspicuously in EG-1, where firmness ranges from roughly 10 to 90 while the median sits near 15. The cause is mechanical rather than analytical. Discrete beads suspended in a semi-solid gel are sampled stochastically by a back-extrusion probe, and each encounter between probe and bead registers as a force spike. These are true measurements and cannot legitimately be excluded as outliers, yet they are heavy-tailed and only weakly coupled to phenolic content, a combination that no linear model handles gracefully and that will re-emerge as a concrete problem in the correlation analysis. Water-holding capacity is the one physicochemical variable with clean group separation, EG-2 reaching 46 to 48% against 38 to 40% for FG-2, which indicates that the beads reinforce serum retention within the casein network. Figure 7 pools the formulations and views the targets by storage day, recovering the Day-7 maximum in distributional form and revealing pronounced bimodality at every time point. The dataset is not one population but two, encapsulated and non-encapsulated, occupying different regions of the response space. Any model required to span both regions must therefore learn a mapping that is locally accurate in two separate neighborhoods rather than one, which raises the bar for a dataset of sixty observations and explains why capacity control became a central concern of the modeling stage.

**Figure 8:**
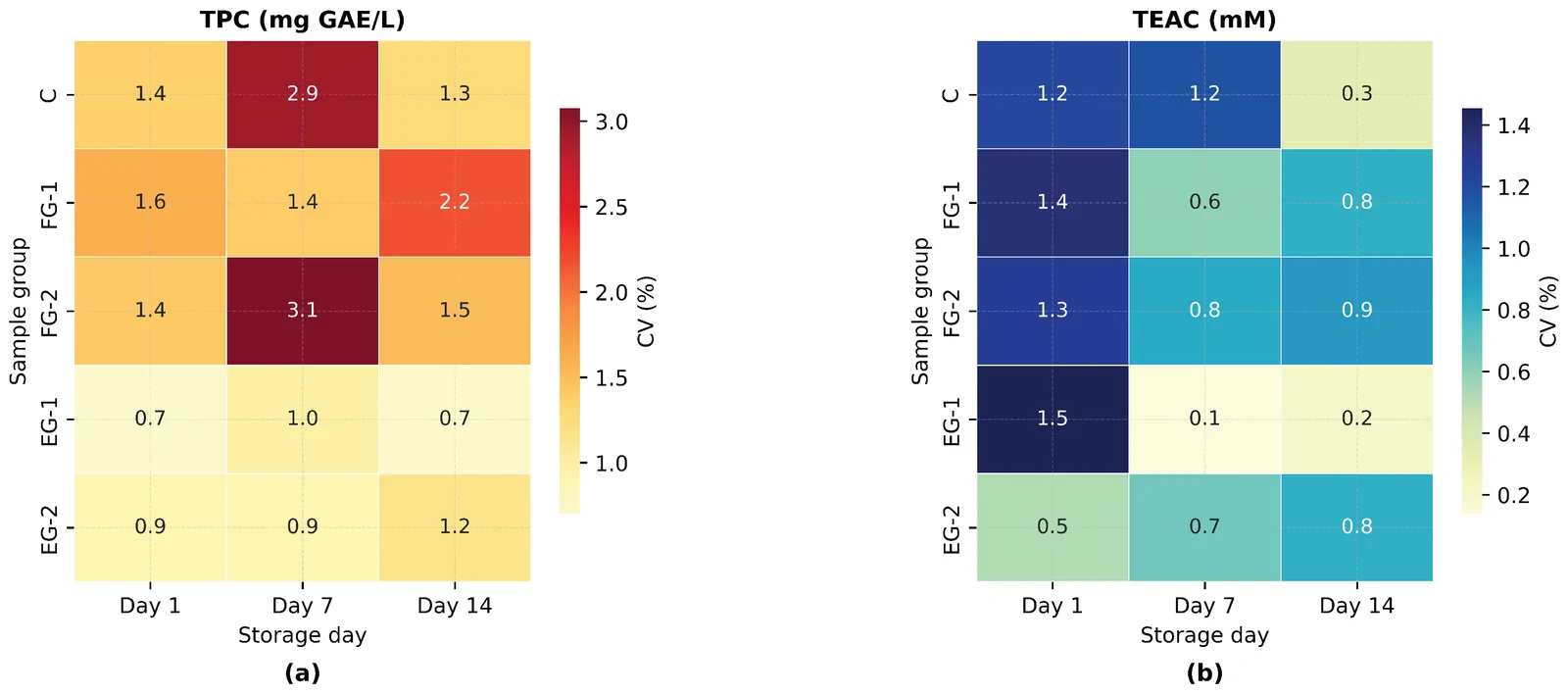
Replicate-level coefficients of variation for (**a**) TPC and (**b**) TEAC by formulation and storage day.

Analytical precision was high throughout and, more importantly, structured. Figure 8 shows that the replicate-level coefficient of variation never exceeded 3.1% for TPC or 1.5% for TEAC, yet it was systematically lower in the encapsulated groups, ranging from 0.7 to 1.0% for EG-1 and from 0.9 to 1.2% for EG-2, against 1.4 to 3.1% for FG-2 and 1.4 to 2.2% for FG-1. Free extract must be dispersed as a solute through a viscous, acidifying medium, and any local heterogeneity in that dispersion surfaces as replicate-to-replicate scatter, whereas beads distribute the payload in discrete, compositionally identical units. Encapsulation thus purchases manufacturing consistency alongside retention. The distinction carries more industrial weight than the mean values alone, because a formulation that meets its label claim reproducibly is worth more than one that meets it on average, and a specification written around a mean with a wide tolerance is a specification that will periodically be missed. Dispersion peaks at Day 7 in the free and control groups, precisely when the phenolic liberation described earlier is most active, and this coincidence is itself corroborating evidence for that mechanism, since a transient chemical process in progress is inherently the least reproducible state at which to sample.

### 4.4. Correlation Structure and the Rationale for Orthogonalization

**Figure 9:**
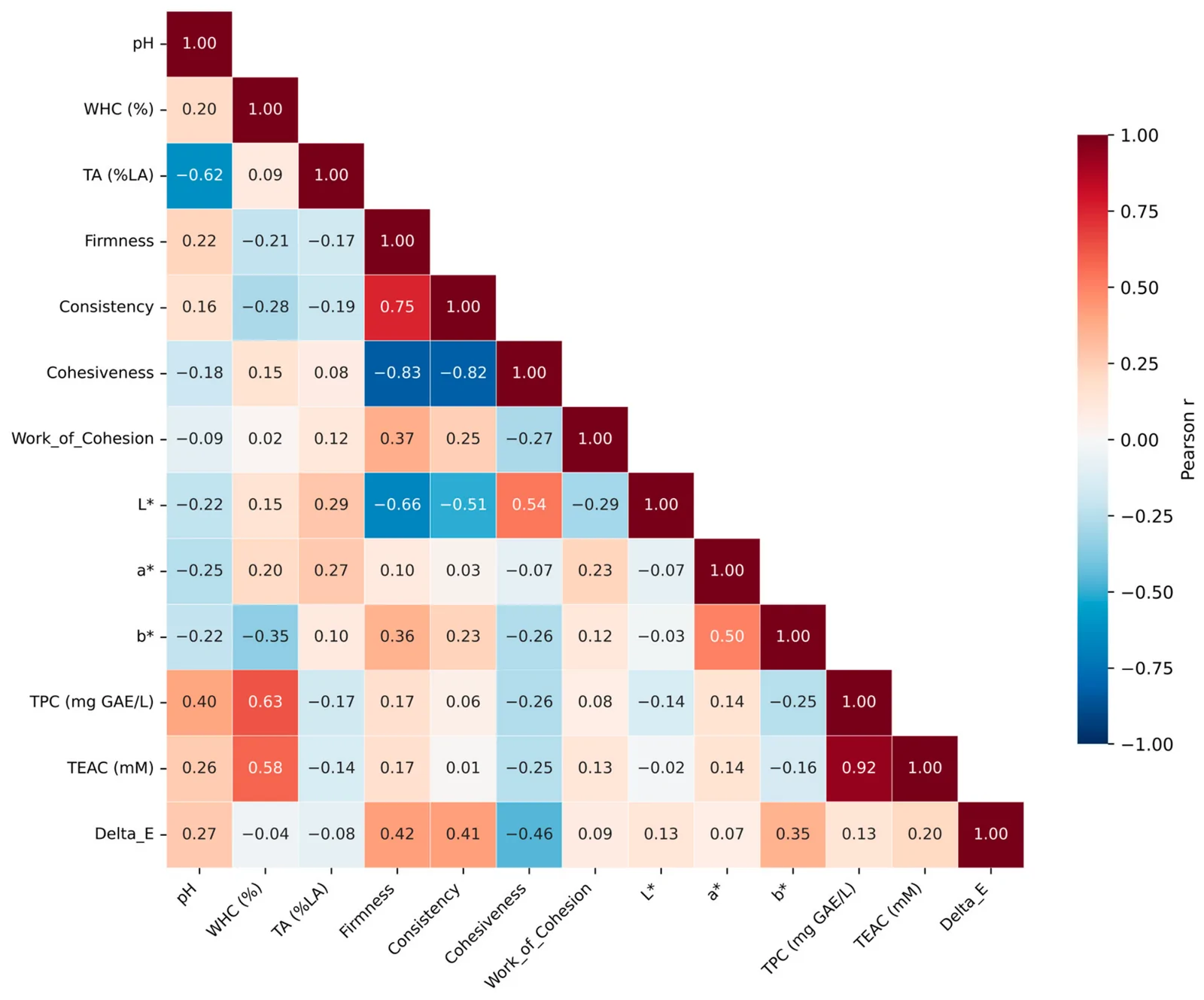
Pearson correlation matrix of the thirteen measured variables.

The heavy-tailed texture variables noted above already hinted that the descriptors are not independent carriers of information, and quantifying their interdependence is the natural next step, since it determines how the panel must be prepared before a network can use it. The linear correlation matrix of Figure 9 makes explicit the redundancy that the modeling pipeline was designed to neutralize. The four textural descriptors form a tightly coupled block, with firmness and consistency, covarying at r=0.75 and cohesiveness opposing both at r=−0.83 and −0.82. They are not four independent probes of the gel but four projections of a single mechanical state and supplying them jointly to a network inflates the condition number of the input Gram matrix without adding information. Lightness carries part of the same signal, and pH and titratable acidity are reciprocal by construction. Two associations bear directly on the prediction problem. TPC and TEAC covary at r=0.92, the strongest coefficient in the matrix, and this coupling is mechanistic, since the species that the Folin reagent quantifies are largely the species that quench the ABTS radical. It is this dependence that justifies predicting both endpoints from a single shared representation rather than training two independent regressors, and the ablation later confirms that the shared encoder is not a convenience but a source of accuracy, since each target constrains the representation the other must use.

**Figure 10:**
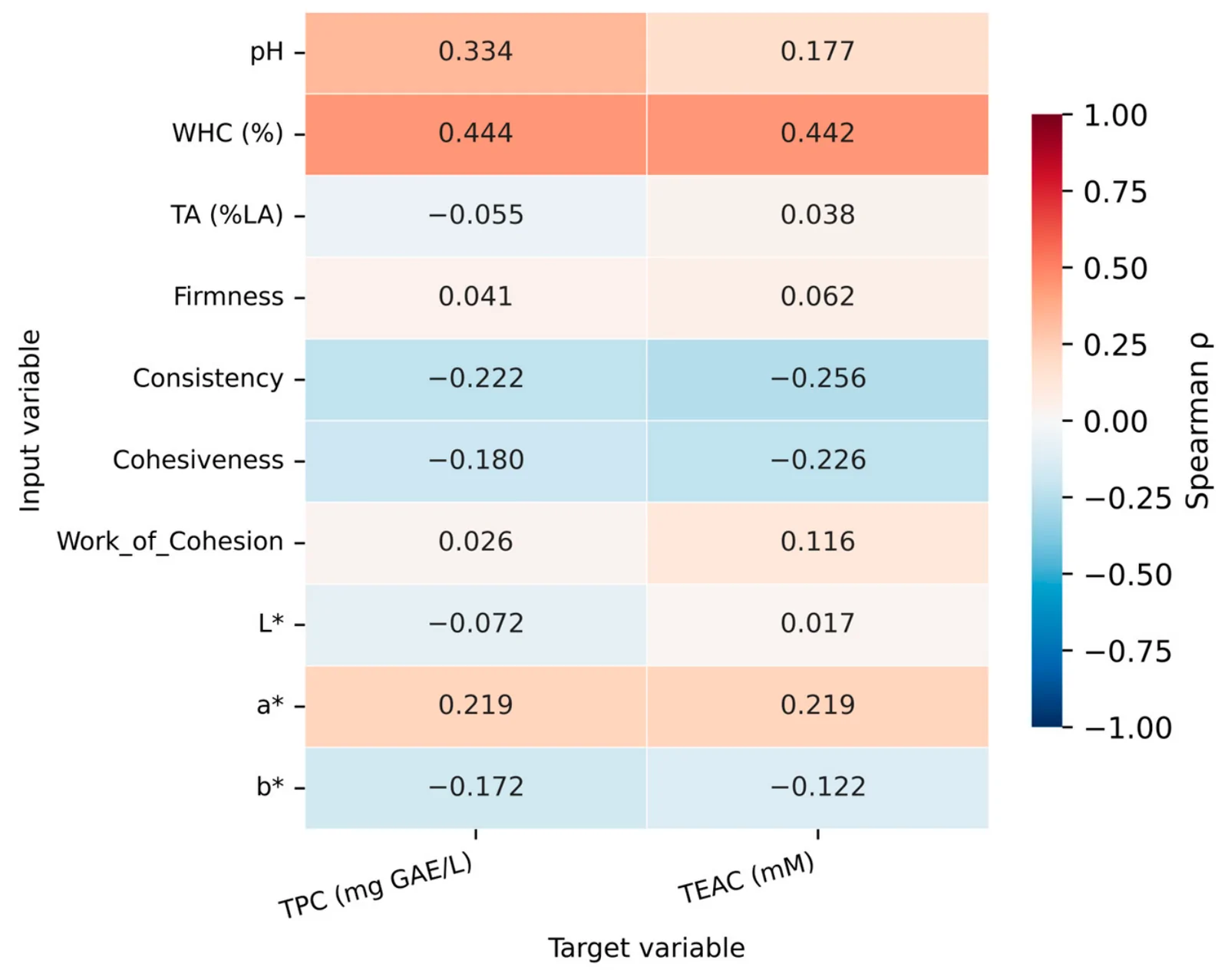
Spearman rank correlation between the ten input descriptors and the two bioactive targets.

Water-holding capacity emerges as the physicochemical descriptor most strongly linked to the bioactive endpoints, at r=0.63 with TPC and 0.58 with TEAC. Grape seed proanthocyanidins bind milk proteins through hydrogen bonding and hydrophobic association, reinforcing the casein network and improving serum retention, so water-holding capacity functions as an indirect, non-destructive read-out of phenolic loading. This is the single most useful finding for the practical ambition of the work, because water-holding capacity is measured by centrifugation and weighing, requires no reagents, and could be automated in a plant far more readily than a colorimetric assay. Figure 10 examines the same relationships on ranks and, in doing so, exposes a trap. Water-holding capacity remains the strongest monotonic correlate of both targets at ρ=0.444 and 0.442, and pH the second for TPC at ρ=0.334, yet the textural variables collapse, with firmness reaching only ρ=0.041 despite its prominence in the linear matrix. The discrepancy between the two coefficients is itself a diagnostic. A descriptor whose Pearson value greatly exceeds its Spearman value owes its apparent linear association to a handful of extreme observations, which is exactly the case for firmness and consistency and exactly what the bead–probe collisions described earlier would predict. Had model selection been guided by the linear matrix alone, the texture block would have been retained as informative, and the network would have been trained to exploit an association that is an artifact of sampling geometry rather than a property of the food.

**Figure 11:**
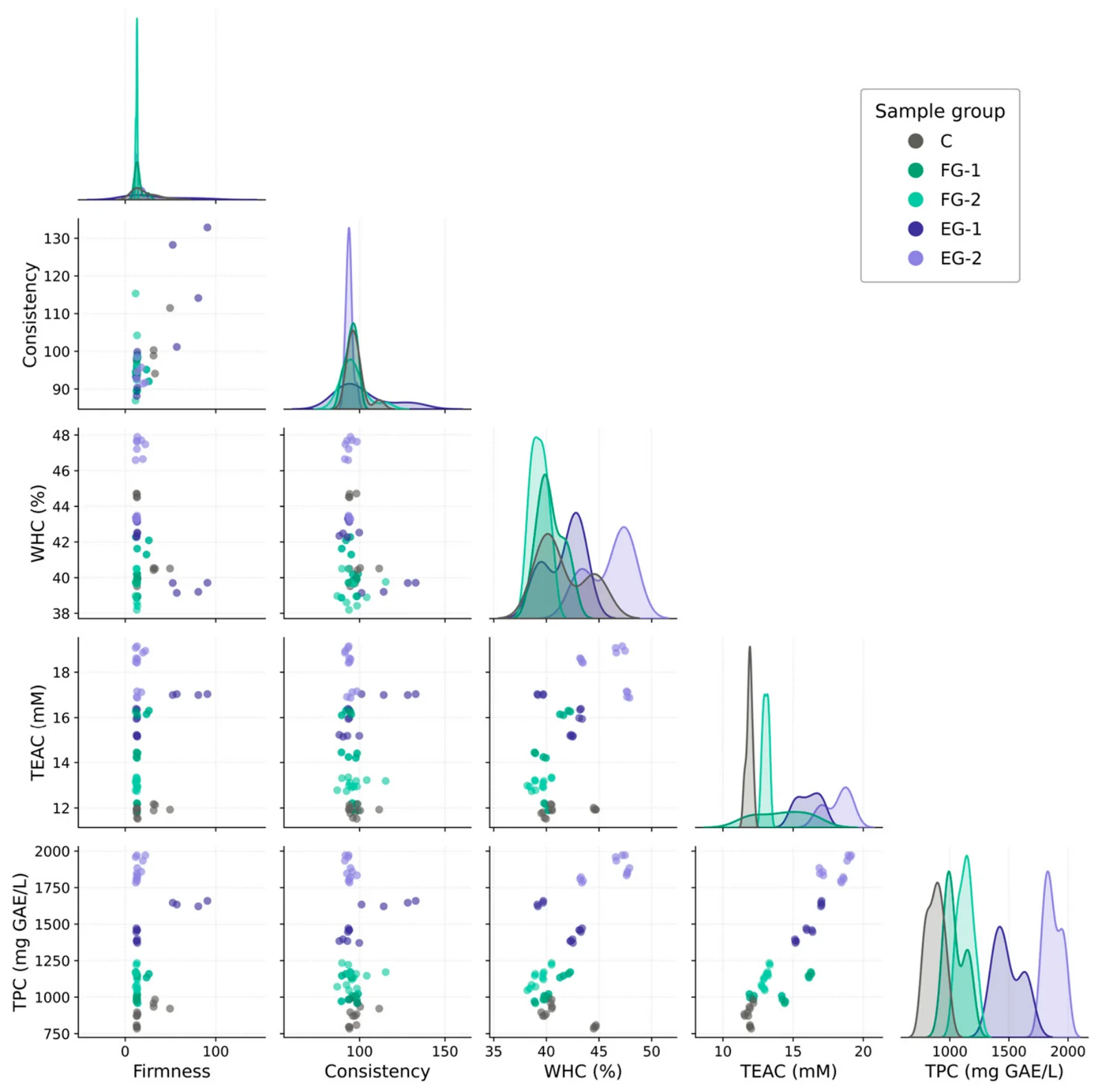
Pairwise scatter and marginal densities of the most informative variables, colored by formulation.

Taken together, the two maps return an unambiguous verdict. No single routine measurement is a sufficient proxy for phenolic content, since the strongest monotonic correlate accounts for under 20% of the rank variance, yet the descriptors are collectively informative and heavily redundant. That combination is the canonical justification for projecting them onto an orthogonal basis before learning, rather than either discarding variables, which would sacrifice the collective signal, or feeding them in raw, which would sacrifice numerical stability. Figure 11 makes the same point geometrically. The formulation clusters resolve cleanly in the planes that pair water-holding capacity and the bioactive targets and superimpose almost completely in the plane that pairs firmness with consistency, so the information the model needs is distributed across the descriptor panel in a way that no single projection recovers.

The marginal densities along the diagonal of Figure 11 sharpen this reading. For both bioactive targets the distributions are clearly polymodal, with the encapsulated formulations forming a well-resolved high-value mode that stands apart from the overlapping control and free-extract populations, whereas the same panels for firmness and consistency collapse into a single narrow peak with a long tail contributed by the bead–probe collisions discussed earlier. A descriptor whose entire between-group signal is confined to a handful of tail observations cannot discriminate formulations reliably, and this is precisely why the texture block, so prominent in the linear correlation matrix, contributes little of predictive value once the analysis moves beyond pairwise coefficients. The contrast on the diagonal thus visualizes, in a single row of panels, the divergence between Pearson and Spearman structures that the correlation maps reported numerically.

More importantly, the off-diagonal scatter reveals that group separability is an inherently multivariate property here rather than the sum of several univariate ones. In the water-holding capacity against total phenolic content plane, the five groups already begin to stratify, but a residual overlap between the control and the lower free-extract formulation persists that neither variable resolves alone; the same overlap closes only when a second, orthogonal direction is admitted. This is the geometric signature of information that lives in the joint distribution rather than in any marginal, and it is the condition under which a linear projection that preserves covariance structure, as principal component analysis does, is expected to outperform any strategy based on ranking or selecting individual descriptors. The pairwise view therefore does more than corroborate the correlation analysis; it demonstrates why the subsequent dimensionality reduction was posed as a rotation of the whole panel rather than as a filter applied to its members, and it anticipates the finding of Section 4.5 that the variable most predictive of the targets loads not on the first component but on the third, where it captures a direction of variation that no single scatter plot in Figure 11 makes fully visible.

### 4.5. Chemometric Feature Extraction

**Figure 12:**
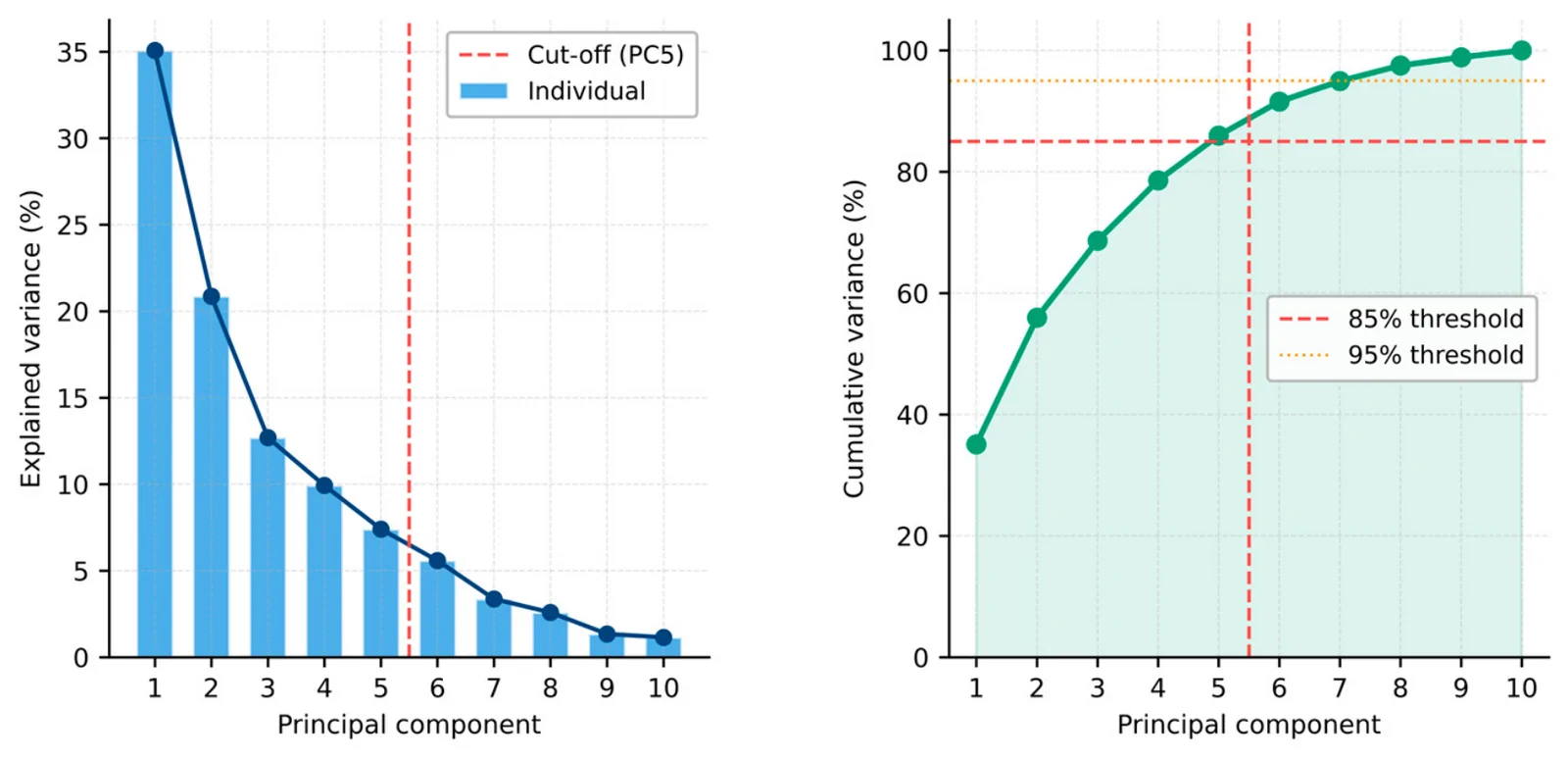
Individual and cumulative explained variance of the ten principal components.

The correlation analysis returned a clear instruction: retain the collective signal of the descriptor panel while removing the collinearity that would destabilize training. Principal component analysis is the transformation that carries out that instruction and applying it to the ten standardized descriptors returned the eigenvalue spectrum shown in Figure 12. The first component absorbs 35.1% of the total variance, and the second a further 20.9%, with a pronounced elbow after the second and a slowly decaying tail beyond it. Five components carry 86% of the variance and satisfy the retention criterion, while a sixth would contribute roughly 5% more. Retaining five halves the input dimension, and the consequence is more than cosmetic for a network that must be fitted to sixty sequences, since every dimension removed is a block of weights that no longer has to be estimated from data incapable of supporting it. The slow decay of the tail itself is worth noting because it indicates that the descriptor panel contains no negligible directions to be discarded costlessly; the reduction achieved here is a considered trade between conditioning and retained information rather than the removal of noise.

**Figure 13:**
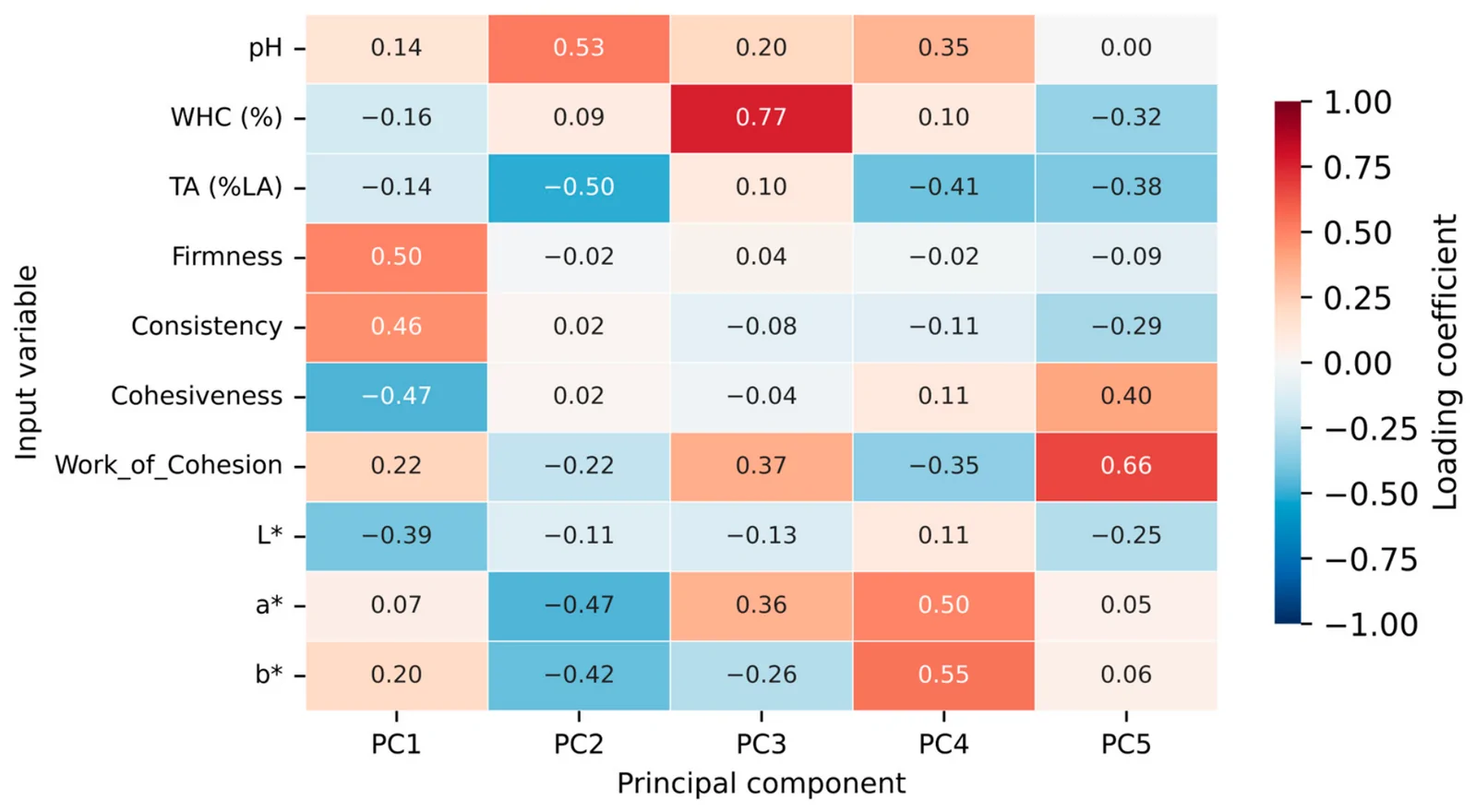
Loadings of the ten descriptors on the five retained principal components.

The loadings presented in Figure 13 show that the retained components are interpretable physicochemical axes rather than statistical abstractions, which is the property that distinguishes a chemometric transformation from an opaque learned embedding. The first component is a pure texture axis, with firmness at 0.50 and consistency at 0.46 opposing cohesiveness at −0.47 and lightness at −0.39, which is exactly the mechanical antagonism visible in the correlation matrix, now compressed into a single coordinate. The second is an acidity and color axis dominated by pH at 0.53 against titratable acidity at −0.50 and the chromatic coordinates. The third is overwhelmingly a moisture axis, with water-holding capacity loading at 0.77, and this carries the sharpest methodological consequence of the analysis. The descriptor most strongly correlated with both bioactive targets is almost absent from the first two components. Had the component count been fixed at two by a naive elbow rule, the single most predictive variable in the panel would have been largely discarded, and the model would have been starved of the very signal the correlation analysis had identified as most valuable. The retention criterion of 85% cumulative variance is what prevented that outcome, and the episode illustrates why a variance-based rule should be preferred to a visual one when the components are subsequently used for prediction rather than for description. The fourth component carries the residual color signal, and the fifth carries the work of cohesion.

Two further features of the loading pattern reward attention. The first is that no descriptor is scattered thinly across all five components; each variable attains a dominant weight on one axis and near-negligible weights elsewhere, so the rotation has produced a nearly simple structure in which every component owns a distinct physicochemical meaning. This separation is what allows the latent inputs to be reasoned about individually and is far from guaranteed, since principal components are constrained only to be orthogonal, not to be interpretable. The second is that the ordering of the components by explained variance does not coincide with their ordering by predictive relevance. The texture axis dominates the variance because the back-extrusion descriptors move over the widest numerical range, yet that range reflects mechanical sampling of the beads rather than phenolic chemistry, whereas the moisture axis, though it accounts for less variance, aligns with the bioactive targets. The analysis therefore separates the direction along which the data vary most from the direction along which they inform the response, and preserving both is precisely why retention was governed by cumulative variance rather than by predictive correlation, which would have privileged the third component and discarded the mechanically dominant but chemically informative first.

**Figure 14:**
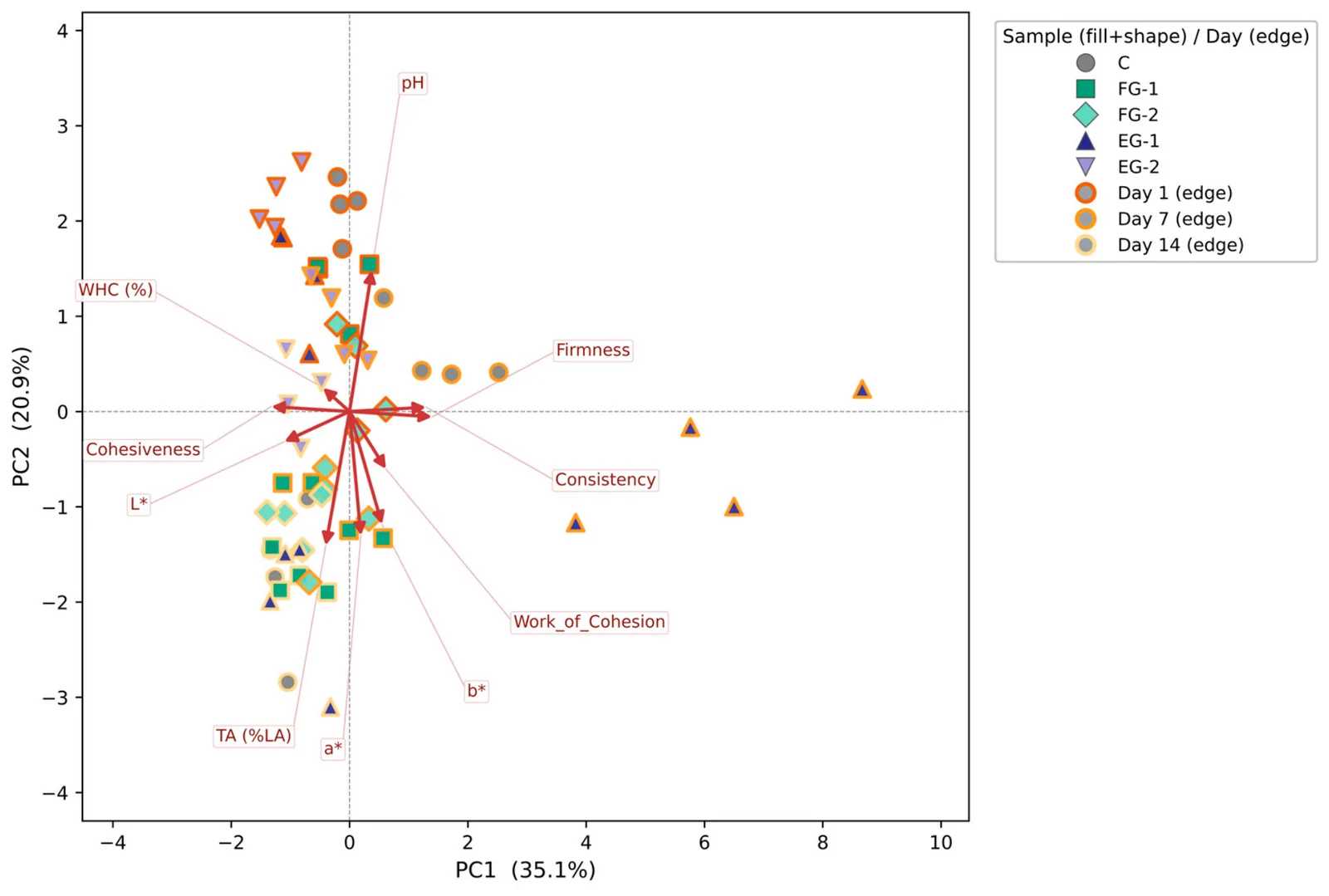
Biplot of the first two principal components with descriptor loadings, colored by formulation and shaded by storage day. Symbols denote the sample groups (C, circle; FG-1, square; FG-2, diamond; EG-1, upward triangle; EG-2, inverted triangle), the marker fill color also encodes the group, and the marker edge color indicates the storage day (Day 1, Day 7, Day 14).

The score plots translate this structure into group behavior. In the plane of the first two components, shown in Figure 14, the control occupies the positive region of the first axis, driven by its firmness and consistency, while the enriched formulations spread along the negative side and separate vertically by acidity and color. Storage day, encoded in marker shading, produces a systematic displacement from Day 1 to Day 14, which establishes that the components capture temporal drift and not merely formulation identity. This is the essential precondition for the sequence encoding that follows, because a representation that separated only the groups would carry no information about how a sample changes and could not support a recurrent model at all. What the biplot demonstrates is that the chemometric axes move with storage in a systematic direction, so the trajectory of a sample through the latent space itself is a meaningful object.

The geometry of that displacement repays closer inspection, because its direction is not shared across the two axes. The storage-related shift occurs predominantly along the second component, the acidity and color axis, rather than along the first, which means that what changes during refrigeration is chiefly the fermentation-driven chemistry of each sample and not its textural identity. A formulation therefore retains its horizontal position, set by the bead-reinforced gel network, while migrating vertically as acidification and color development proceed, and this decoupling of the temporal direction from the group direction is exactly the property a recurrent model can exploit, since it can learn the trajectory without having to disentangle it from the far larger between-group separation. Had storage instead moved samples along the same axis that distinguishes the formulations, the two sources of variation would have been confounded in the leading component, and no amount of sequence modeling could have separated a change in time from a difference in recipe.

A second feature is the unequal length of the trajectories. The encapsulated formulations trace visibly shorter paths through the plane than the control and the free-extract groups, a geometric restatement of the stability already seen in the raw storage curves: samples whose bioactives are protected drift less far in chemometric space because their underlying physicochemistry is changing more slowly. The score plot thus encodes, in the length of a path rather than the position of a point, the same shelf-life advantage that the trajectory plots reported directly. This convergence of two independent representations on the same conclusion strengthens confidence that the latent space is tracking a real physical process, and it clarifies why the network was given the ordered sequence of scores rather than a single pooled descriptor: the informative quantity is not where a sample sits at any one time but the direction and distance it travels between successive storage points.

**Figure 15:**
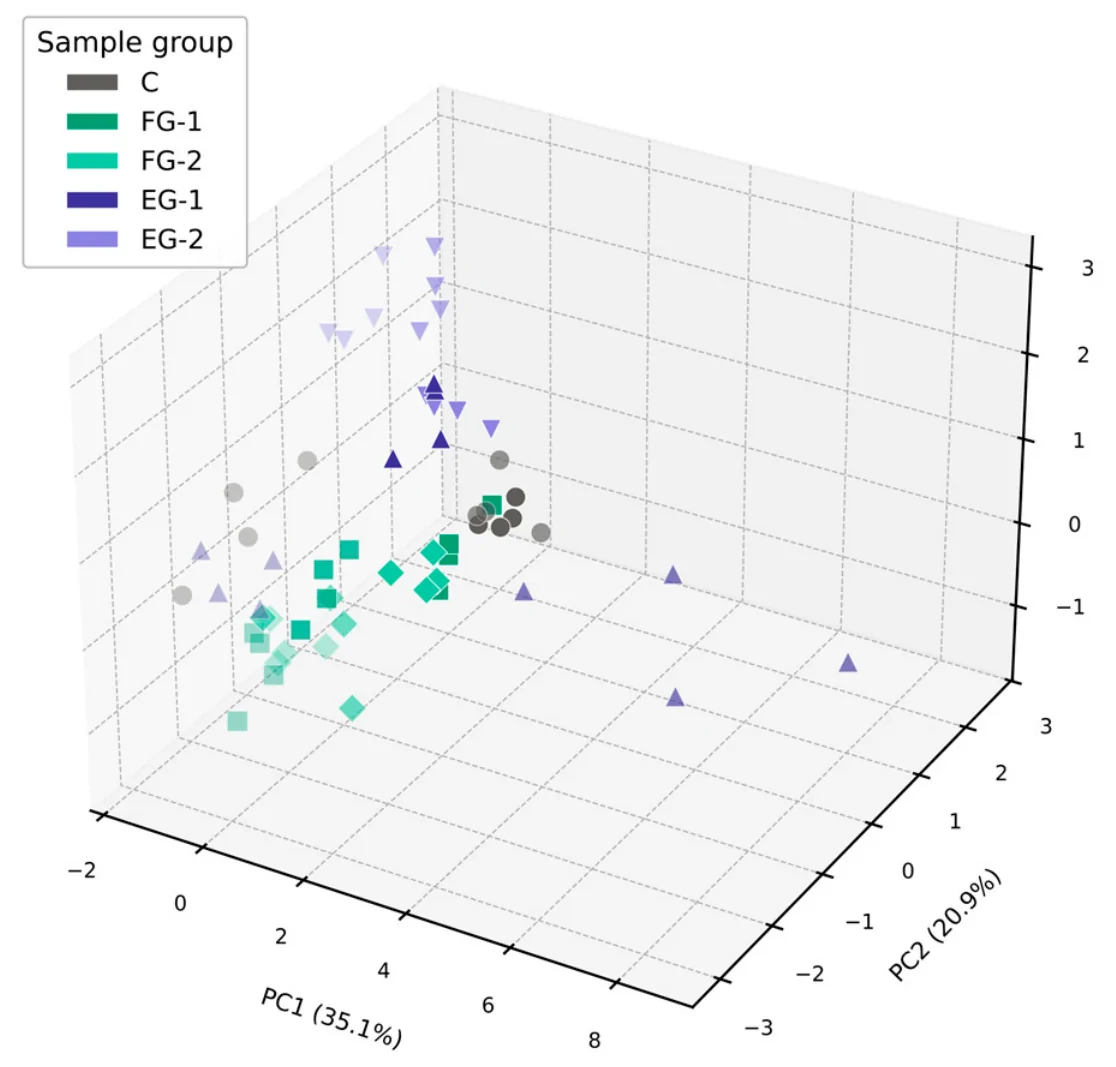
Score plot in the space of the first three principal components. Symbols denote the sample groups: C, grey circles; FG-1, green squares; FG-2, light-green diamonds; EG-1, dark-blue upward triangles; EG-2, purple downward triangles. The three axes are the first three principal components (PC1, PC2 and PC3), with the percentage of variance explained given on each axis.

Adding the moisture-dominated third component, as in Figure 15, resolves EG-1 and EG-2 into distinct clouds that overlap in two dimensions. The third axis therefore carries formulation information that the leading pair does not, which is the geometric counterpart of the loading result and an independent confirmation that the five-component retention was necessary rather than conservative.

The separation recovered along the third axis is not arbitrary but follows the encapsulation dose, since EG-1 and EG-2 differ only in the quantity of beads they carry, and it is water-holding capacity, the variable that loads most heavily on this component, that responds to that difference. The three-dimensional view therefore does more than add visual clarity; it shows that the very direction a two-component projection would discard is the one that separates the two formulations of greatest interest, namely the high-performing encapsulated products whose antioxidant claim a manufacturer would most want to certify. A reduction stopped at two components would have collapsed precisely the distinction on which the commercial value of the work rests, and the fact that this distinction reappears on the third axis rather than being lost altogether is what justifies describing the retained representation as complete for the present purpose.

This observation also reframes how the moisture axis should be understood in the modeling that follows. Because water-holding capacity is both the strongest correlate of the bioactive targets and the descriptor that resolves the encapsulated formulations, the third component is doing double duty: it carries formulation identity and predictive signal on the same coordinate, and the two roles reinforce rather than compete. A model supplied with this component receives, in a single input dimension, information that is simultaneously discriminative between recipes and informative about their phenolic load. The economy of that encoding is one reason the network attains high accuracy from a modest number of latent inputs, since the retained components are not merely orthogonal but individually purposeful, each contributing a physically distinct and non-redundant piece of the mapping from physicochemistry to antioxidant status. The three-dimensional score plot thus closes the chemometric analysis by demonstrating that dimensionality was reduced without discarding any direction the prediction task requires.

### 4.6. Hyperparameter Optimization and Training Behavior

The input representation and the storage-time tensor were settled before any tuning began, which left the network’s own configuration as the only remaining unknown. Six of its parameters take no value that theory can prescribe, and these were fixed by minimizing the criterion given earlier in Section 3.8 as Equation (30), the three-fold cross-validated mean squared error with $K_{cv}=3$, computed on the scaled targets and confined to the training partition. Two aspects of that criterion set the limits of what the tuning can be said to establish. Because the folds are cut solely from the training data, no held-out sample ever enters the selection, and the accuracy carried forward to Section 4.7 is a true out-of-sample quantity rather than a number the search could have inflated upward. Since the targets are scaled before the norm is applied, for the reason already given in Section 3.8, the phenolic response, larger by two orders of magnitude, is prevented from commandeering the loss and skewing the fit toward TPC while TEAC is left poorly served. The rival optimizer met the identical objective, domain, and iteration count, so that the contrast drawn in Section 4.8 rests on search behavior alone.

**Figure 16:**
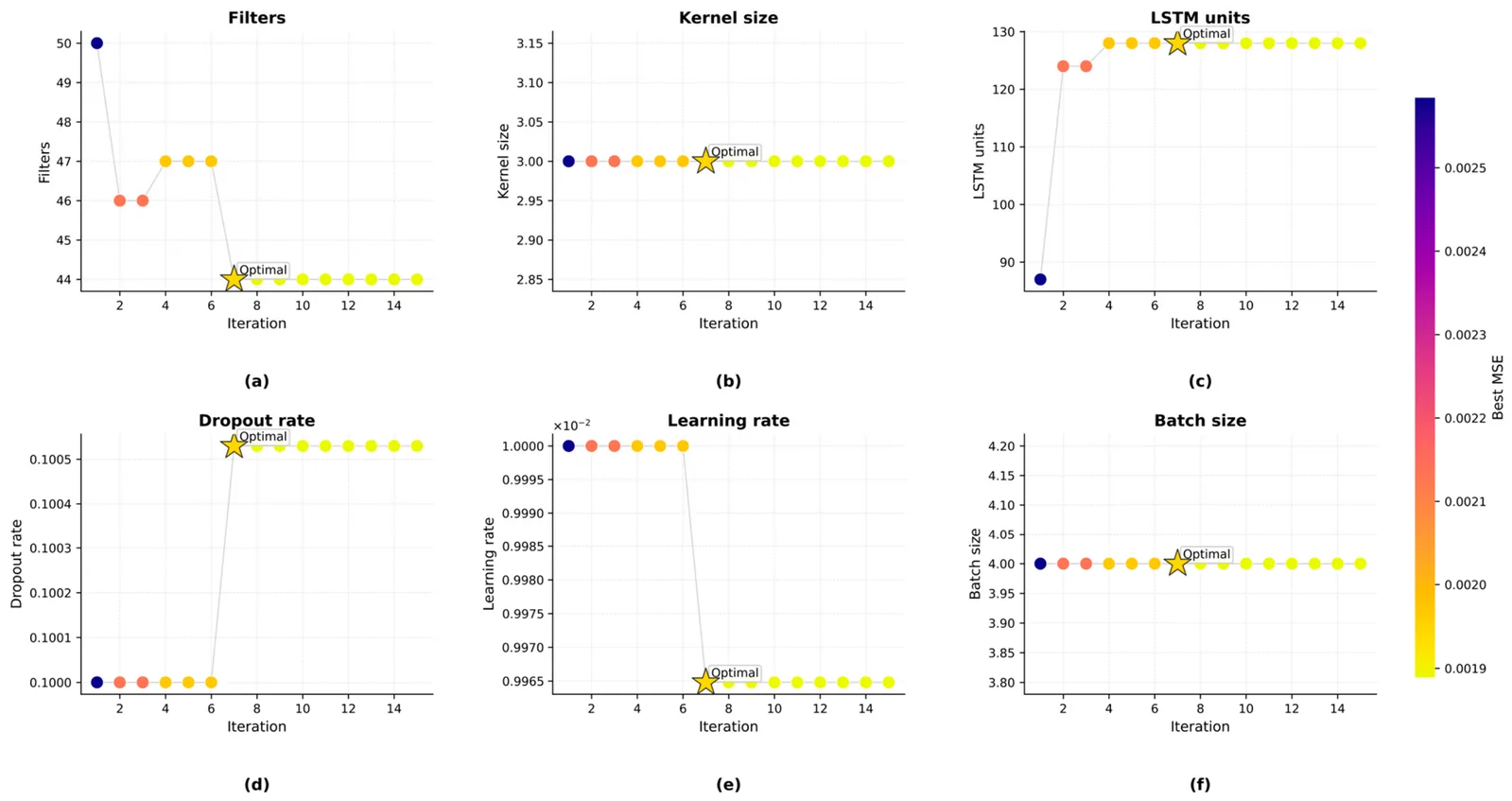
Iteration-wise trajectory of the six tuned hyperparameters during the CPO search, with each point colored by the best cross-validated mean squared error (MSE) and the optimum marked by a star: (**a**) number of Conv1D filters; (**b**) kernel size; (**c**) LSTM units; (**d**) dropout rate; (**e**) learning rate (ordinate scaled by ×10^−2^); and (**f**) batch size.

As Figure 16 shows, the run came to rest at 44 convolutional filters, a kernel three units wide, 128 LSTM units, dropout at 0.100, a learning rate of $9.965\times 10^{-3}$, and a batch size of 4, with the cross-validated error holding near $1.9\times 10^{-3}$. That minimum surfaced at the seventh of fifteen iterations and stood unchallenged thereafter, having fallen from about $2.6\times 10^{-3}$ at initialization. Settling in half the available iterations is what cyclic population reduction buys in practice, since retiring the poorest agents early lets the surviving trials gather over the promising region rather than re-testing ground already known to fail, an economy that tells when each fitness call means training a network end to end.

**Figure 17:**
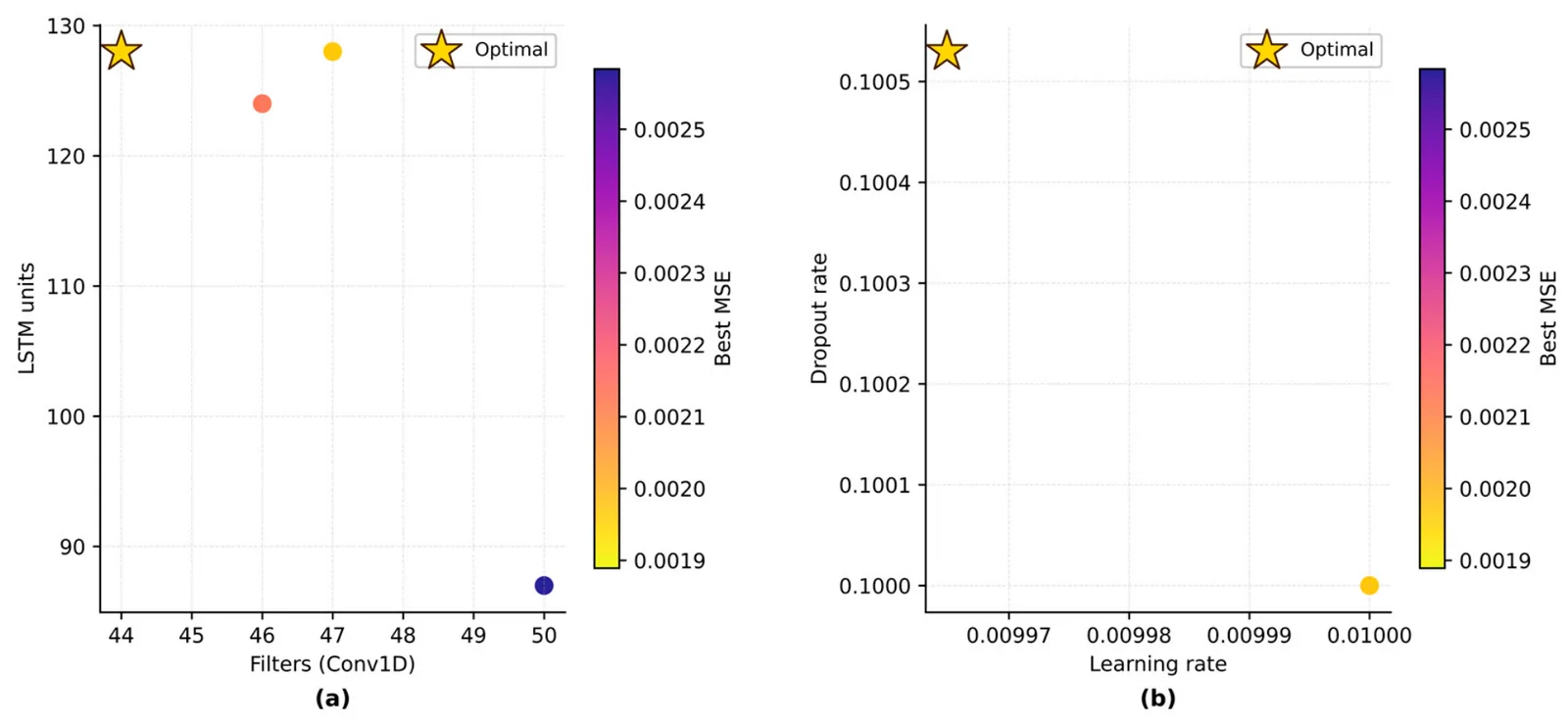
Explored hyperparameter space in the (**a**) filter and LSTM unit plane and the (**b**) learning rate and dropout plane, colored by best cross-validated MSE.

The configuration that emerged merits a closer reading. Recurrent capacity was carried to the upper edge of its range while the filter count was left mid-range at 44, an allocation that seats the predictive work in temporal memory rather than local feature extraction. For a three-step sequence whose informative content lies in the trajectory and not in any single day, that outcome is the natural one, and it reinforces from an independent direction the decision to treat storage day as a sequential axis. Both dropout and batch size came to rest at the bottom of their ranges, and the two settled the same argument. Across sixty training sequences, forceful stochastic regularization would sacrifice signal the model cannot replace, while small batches furnish the frequent, noisy gradient steps that regularize instead. Figure 17 casts the sampled space onto two diagnostic planes, and the optimum is seen to occupy a fitness pocket set clearly apart from its neighbors, a sign that the search told strong configurations from weak ones rather than sliding across a flat surface where any choice would have sufficed.

**Figure 18:**
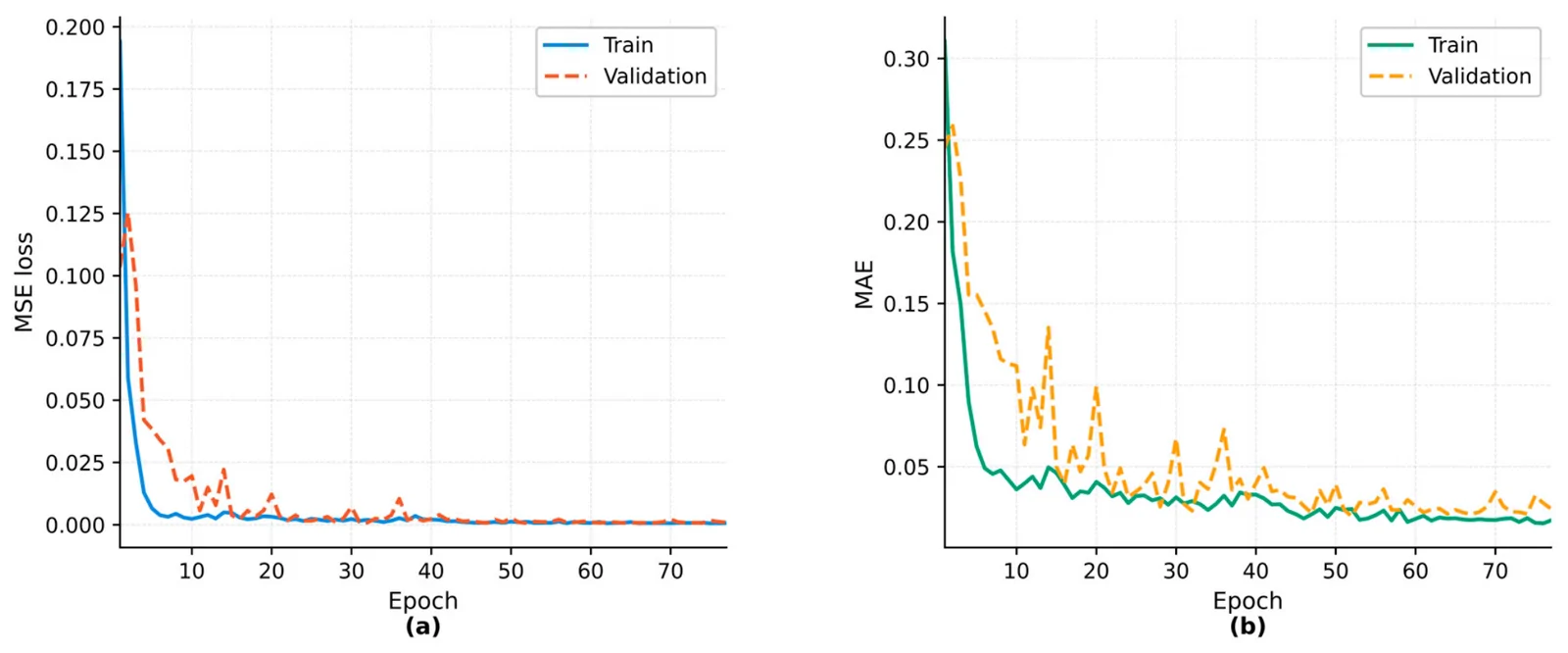
Training and validation trajectories of (**a**) MSE loss and (**b**) MAE for the CPO-optimized model.

Training the selected network ran its course without pathology. In Figure 18 the loss and the mean absolute error drop steeply over the opening ten epochs and then flatten across roughly seventy-seven, the validation curves keeping pace with their training counterparts and never bending upward in the manner that would announce memorization. For a model raised on sixty augmented sequences, this single observation outweighs any accuracy figure, since a network of comparable capacity could in principle reproduce its training set wholesale and post a creditable validation score purely by how the split happened to fall. That no divergence appears is what confirms that chemometric compression, dropout, early stopping, and bounded augmentation jointly held the network within a capacity the data can genuinely bear. What remains of the oscillation in the validation curve is simply the mark of a small validation set, where one sequence more or less noticeably tilts the meaning, and it carries no implication of instability.

### 4.7. Predictive Performance and Diagnostic Validation

The configuration selected and trained in the preceding subsection had, until this point, seen only the training partition; its value is decided by how it performs on the samples withheld from that process. On the five held-out sequences, the CPO-optimized model reached an RMSE of 41.04 mg GAE/L, an MAE of 32.14 mg GAE/L, a MAPE of 2.82%, and an $R^{2}$ of 0.988 for TPC, together with an RMSE of 0.352 mM, an MAE of 0.275 mM, a MAPE of 1.91%, and an $R^{2}$ of 0.959 for TEAC, as summarized in Table 4. Two comparisons give these numbers meaning. The phenolic error amounts to roughly 3% of a mean concentration exceeding 1200 mg GAE/L, which is the same order as the replicate-level analytical dispersion reported in Figure 8, so the uncertainty of the model has converged towards the precision of the assay it is intended to replace. Beyond that point further accuracy would be difficult to demonstrate because the reference measurement itself is not more reproducible. In addition, a MAPE below 10% is conventionally taken as the threshold for good predictive accuracy in applied food modeling [59], and values of 2.82% and 1.91% clearly beat that benchmark by a wide margin.

**Figure 19:**
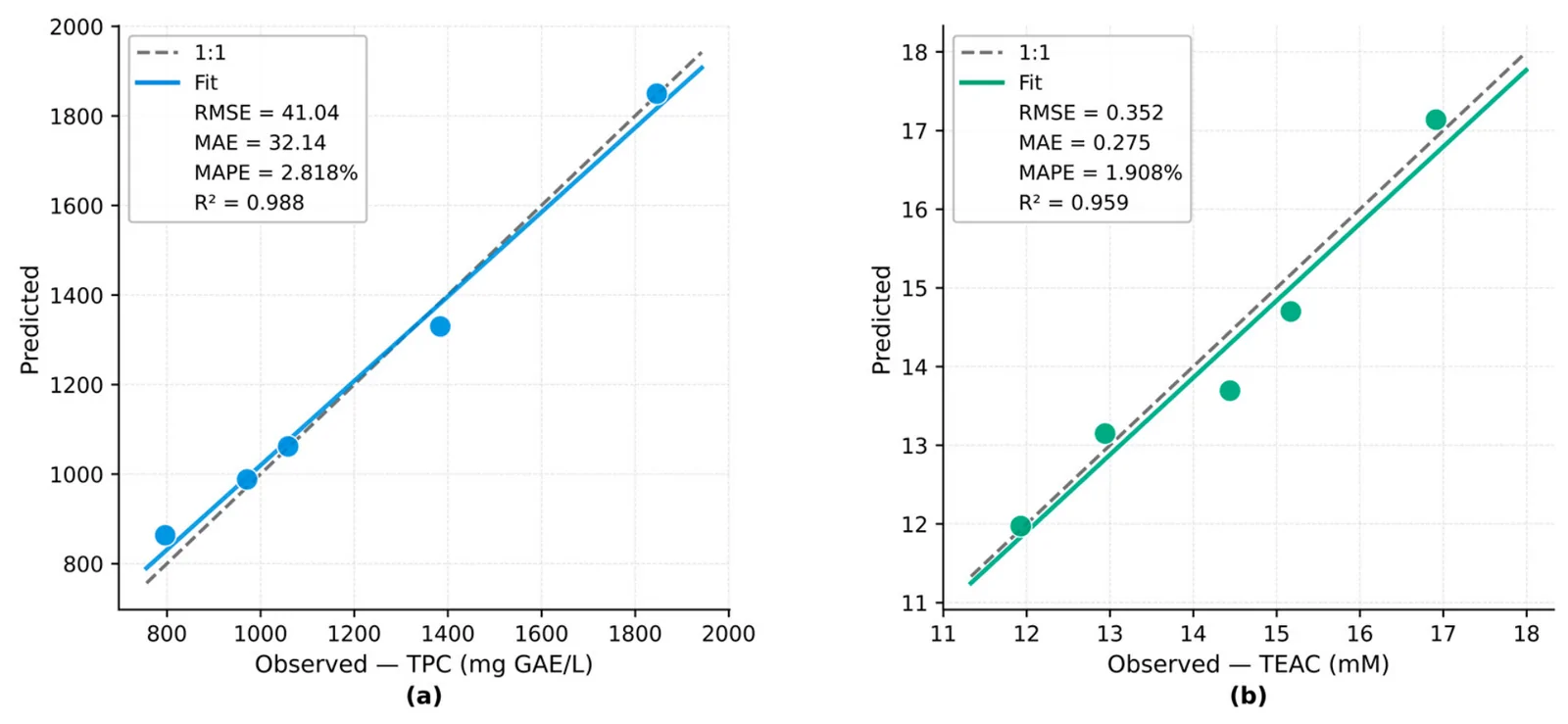
Observed versus predicted values on the held-out test set for (**a**) TPC and (**b**) TEAC.

Figure 19 plots the predictions against the assayed values and shows them hugging the identity line from the control near 800 mg GAE/L to EG-2 near 1850 mg GAE/L, so the accuracy is not an artifact of interpolating a narrow band. This span is the relevant test, because the five held-out sequences were chosen to represent each formulation and therefore traverse the full concentration range the product family occupies. A model that performed well only within the crowded lower half of that range would be of little use for the encapsulated formulations, which are precisely the ones whose antioxidant claim would be made on a label.

Two further features of the scatter deserve comment. The points show no fanning as concentration rises, which means the prediction variance stays roughly constant rather than swelling at the upper end where the encapsulated samples sit, and a fitted line through the cloud would carry a slope close to unity with an intercept near the origin, so the model neither compresses the high values nor lifts the low ones. Both traits matter for the intended use. A quality control decision made at the labeling threshold depends on the estimate being unbiased at that particular level, not merely on average across the range, and a slope that drifted below one would understate exactly the formulations engineered to be most potent. The agreement seen across the whole interval therefore speaks to the practical question the assay is meant to settle, not only to a global goodness of fit that a few dense clusters could otherwise satisfy.

**Figure 20:**
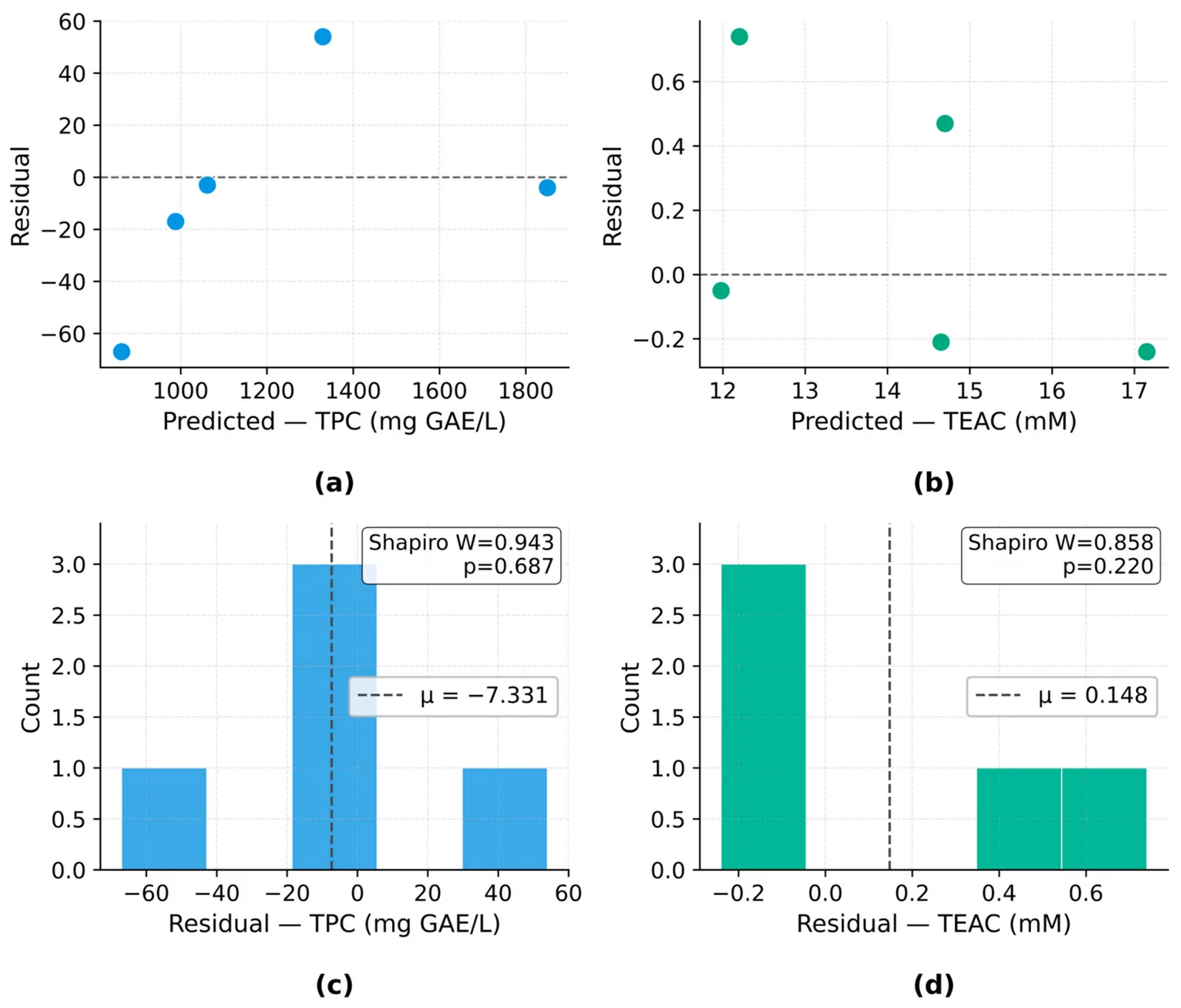
Residuals against predicted values and residual distributions for (**a**,**c**) TPC and (**b**,**d**) TEAC.

Strong aggregate metrics can nevertheless conceal a model that is wrong in a structured way, and the robustness of the proposal rests less on the numbers above than on the diagnostics that follow. In Figure 20 the residuals scatter about zero with no discernible trend against the predicted value, which excludes the most common failure mode of a regressor fitted to a stratified dataset, in which predictions drift high at low concentrations and low at high ones. Their distributions are centered near zero, the mean residual of −7.33 mg GAE/L for TPC amounting to 0.6% of the mean concentration, and the Shapiro–Wilk test does not reject normality for either target, returning *W* = 0.943 with *p* = 0.687 for TPC and *W* = 0.858 with *p* = 0.220 for TEAC.

The two targets are worth reading separately here. The lower W for TEAC reflects its smaller sample and wider spread rather than any genuine departure, since its *p* value stays well above the conventional threshold and the normal quantile plot bends only slightly at the tails. This distinction matters because a formal rejection would have cast doubt on any interval built from the errors, whereas the result obtained leaves that inference intact. That the residuals behave as noise and not as an unmodeled signal still awaiting a functional form is ultimately what licenses treating the reported confidence bounds as trustworthy rather than provisional.

**Figure 21:**
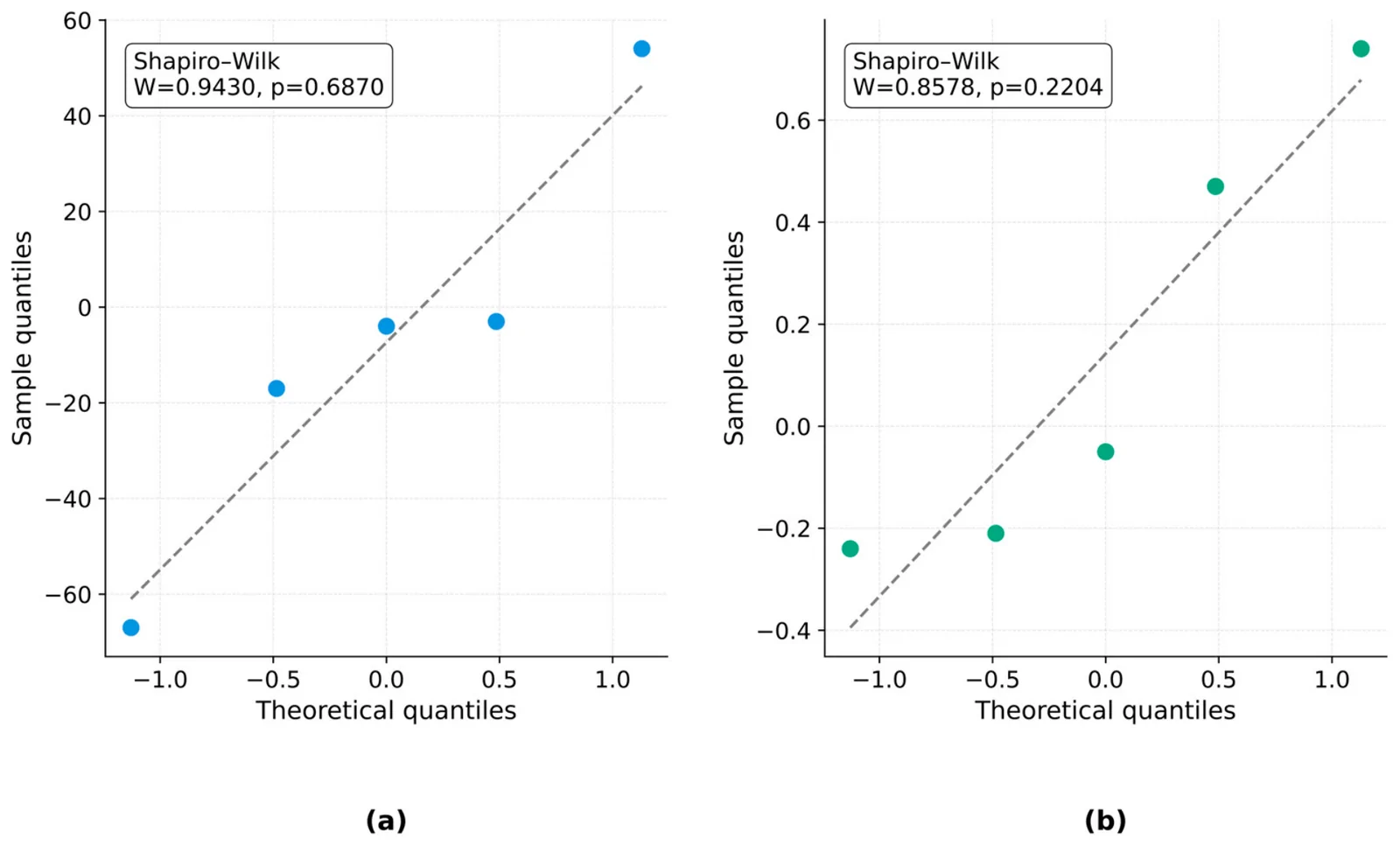
Normal quantile–quantile plots of the residuals for (**a**) TPC and (**b**) TEAC, with the Shapiro–Wilk statistic.

The quantile–quantile plots of Figure 21 present the same evidence graphically, with the residuals falling close to the theoretical line for both endpoints. The claim these statistics support is not that the residuals are normal in some deep distributional sense, since with five test points no such claim could be sustained and the power of the test is correspondingly low. It is rather that what survives prediction is unstructured scatter rather than a learned, deterministic component of the storage kinetics. Had the model failed to capture, for instance, the depolymerization event that distinguishes FG-1, that failure would appear as a residual whose sign is systematic within a formulation, and neither the residual plots nor the normality tests give any indication of such structure.

**Figure 22:**
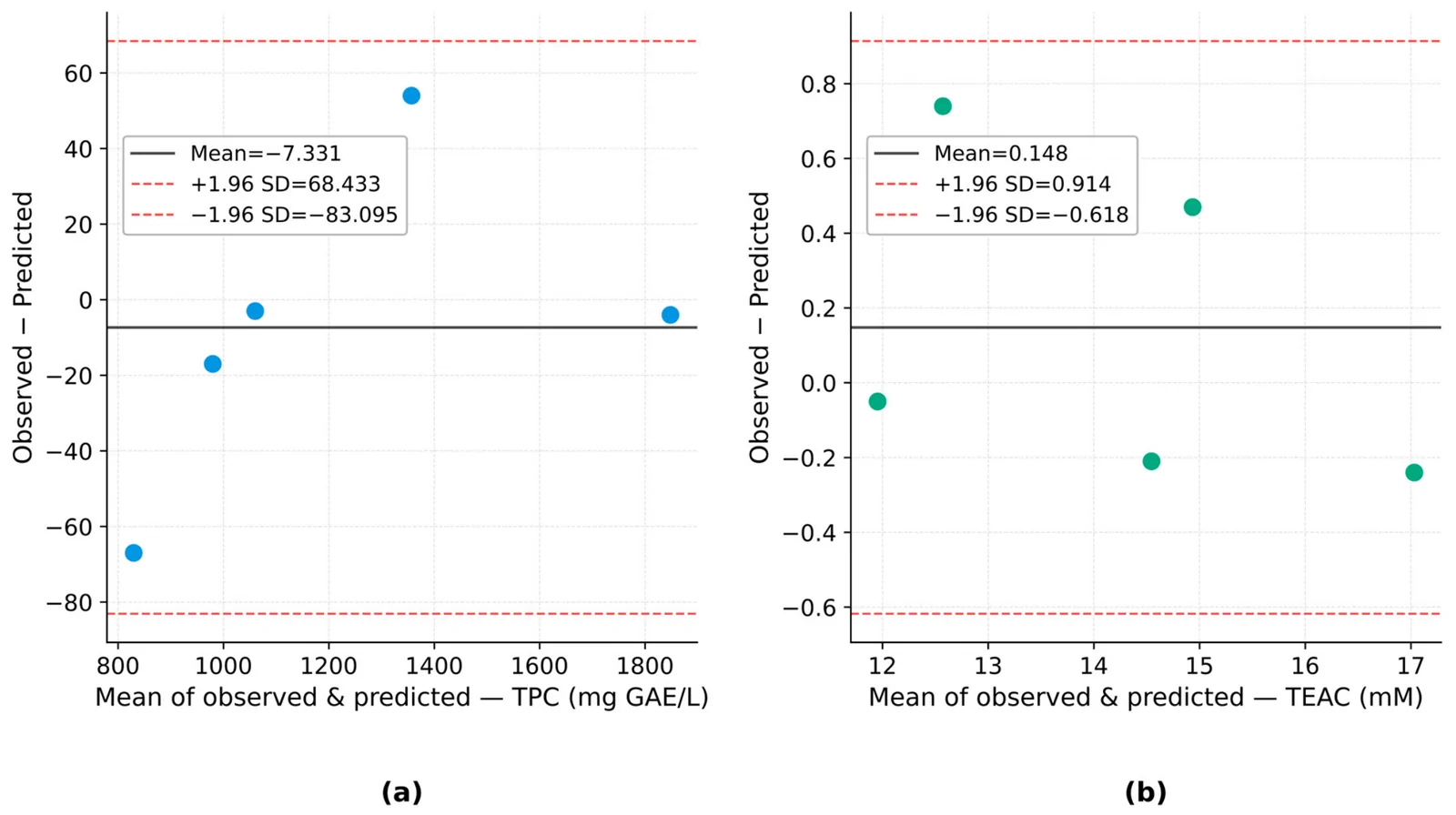
Bland–Altman agreement between predicted and assayed values for (**a**) TPC and (**b**) TEAC.

The agreement analysis of Figure 22 addresses the question on which the practical value of the framework turns; namely, whether the model may stand in for the assay rather than merely resemble it. For TPC the mean difference is −7.33 mg GAE/L with 95% limits of agreement spanning −83.10 to +68.43 mg GAE/L, while for TEAC the mean difference is +0.148 mM with limits from −0.618 to +0.914 mM. Both intervals are narrow relative to the ranges they span, and neither displays a proportional trend, as the differences do not widen when concentration rises. This is the decisive result. A model may post a low RMSE and still drift systematically at high concentrations, in which case it could never certify a high-antioxidant product, because its error would be largest precisely where the label claim is made. The absence of such drift, combined with a mean difference well below 1% of the target value, is what licenses the framework as a substitute for the Folin–Ciocalteu and ABTS assays rather than merely as a well-fitting regressor.

The limits of agreement carry a further implication for how the substitution would operate in practice. Because they bound the disagreement a single prediction may show against the reference, a laboratory can weigh that width directly against its own tolerance for a given decision. Where the interval sits inside the margin a specification allows, the model may replace the wet assay outright, and where it does not, it can still triage samples so that only the borderline cases proceed to full titration, which is where most of the analytical cost is incurred. A boundary on this substitution should also be noted: because the model was calibrated on discrete storage points under controlled refrigeration, it describes an isothermal shelf-life trajectory, and extension to active fermentation or a fluctuating cold chain would require appending the corresponding time points and time–temperature covariates to the input sequence, which is left to future work.

### 4.8. Ablation Analysis

**Figure 23:**
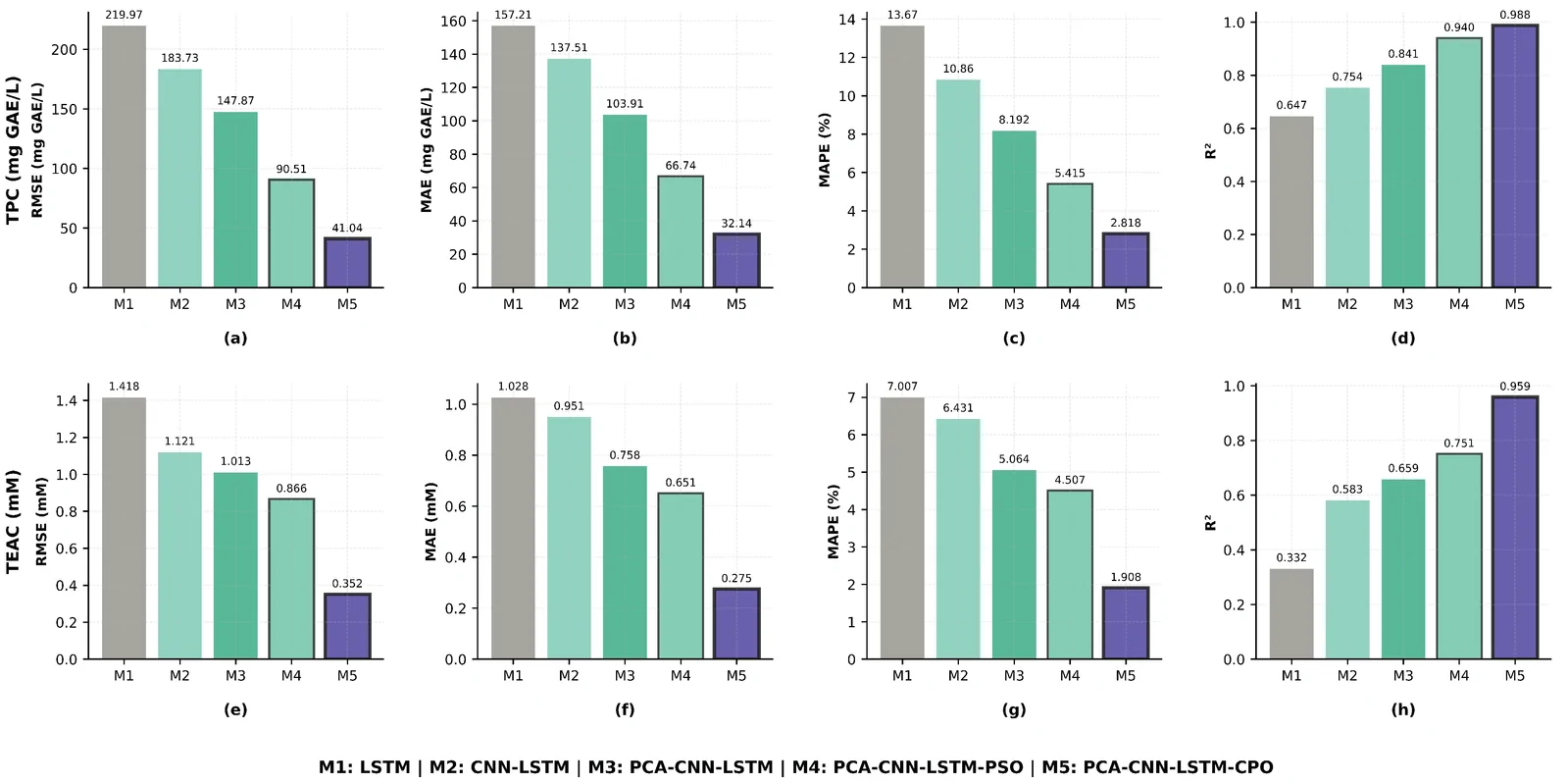
Ablation results for TPC in (**a**–**d**) and TEAC in (**e**–**h**) across the five configurations.

The diagnostics established that the complete framework predicts accurately and without systematic bias, but they say nothing about which of its parts are responsible, and a pipeline that combines chemometric reduction, convolution, recurrence, and metaheuristic tuning invites the suspicion that some of these are redundant. The ablation settles the question directly. Table 5 and Figure 23 record a monotonic improvement across all four metrics and both targets as components are introduced, which is the strongest available evidence that no element of the pipeline is decorative. For TPC the RMSE falls from 219.97 mg GAE/L for the recurrent baseline to 41.04 mg GAE/L for the full model, a reduction of 81.3%, while $R^{2}$ rises from 0.647 to 0.988. For TEAC the RMSE falls from 1.418 to 0.352 mM, and $R^{2}$ climbs from 0.332 to 0.959. The interpretation lies in the step-by-step decomposition rather than in the endpoints.

Adding the convolutional block to the recurrent baseline reduced the TPC RMSE by 16.5%. Thus, even across a three-step sequence, explicitly extracting transitions between consecutive measurement days provides information that the recurrent layer does not fully capture on its own. The improvement is meaningful but moderate, as would be expected when the temporal dimension is short, and it would therefore be misleading to present the convolutional block as the decisive component of the architecture.

Introducing the chemometric front-end reduced the RMSE by a further 19.5%. This is also the component whose contribution might be most readily questioned a priori, since a projection retaining 86% of the variance necessarily discards the remaining 14%. The ablation results directly address this concern by suggesting that the discarded variation primarily reflects redundancy rather than useful predictive information. As demonstrated by the correlation matrix, the textural descriptors are so strongly intercorrelated that they introduce collinearity rather than independent information. Removing this collinearity improves the numerical conditioning of the input space and stabilizes the gradient estimates on which model training depends. For a small, strongly correlated food dataset, the conditioning benefit provided by orthogonalization may therefore outweigh the nominal loss of variance. This finding may also extend beyond the present dataset, as highly collinear descriptor panels are common in food analysis.

Hyperparameter optimization delivered the largest performance gains. Replacing the default settings with PSO-based tuning reduced the TPC RMSE by 38.8%, confirming that off-the-shelf architectures were substantially suboptimal for this task. This is an important point to note, as default configurations are frequently reported in the food modeling literature without adequate justification. Replacing PSO with CPO produced a further 54.7% reduction in TPC RMSE and a 59.4% reduction in TEAC RMSE, while the corresponding MAPE values decreased from 5.42% to 2.82% and from 4.51% to 1.91%, respectively. The markedly different outcomes obtained by two optimizers searching the same domain, using the same objective function and evaluation budget, constitute the central methodological finding of this study. Since all other experimental conditions were held constant, the observed difference may be attributed to the search behavior of the optimizers, particularly the cyclic population reduction mechanism employed by CPO. A fixed-size swarm may spend its later evaluations reassessing particles that have already converged toward the global-best region, thereby yielding diminishing information from each fitness evaluation. By contrast, the population reduction schedule removes less productive agents and reallocates the remaining computational effort to regions in which further improvement is still possible. Under a severely constrained fitness evaluation budget, which is the norm rather than the exception when each evaluation requires training a neural network, this reallocation may provide greater benefit than further refinement of the update rule itself.

**Figure 24:**
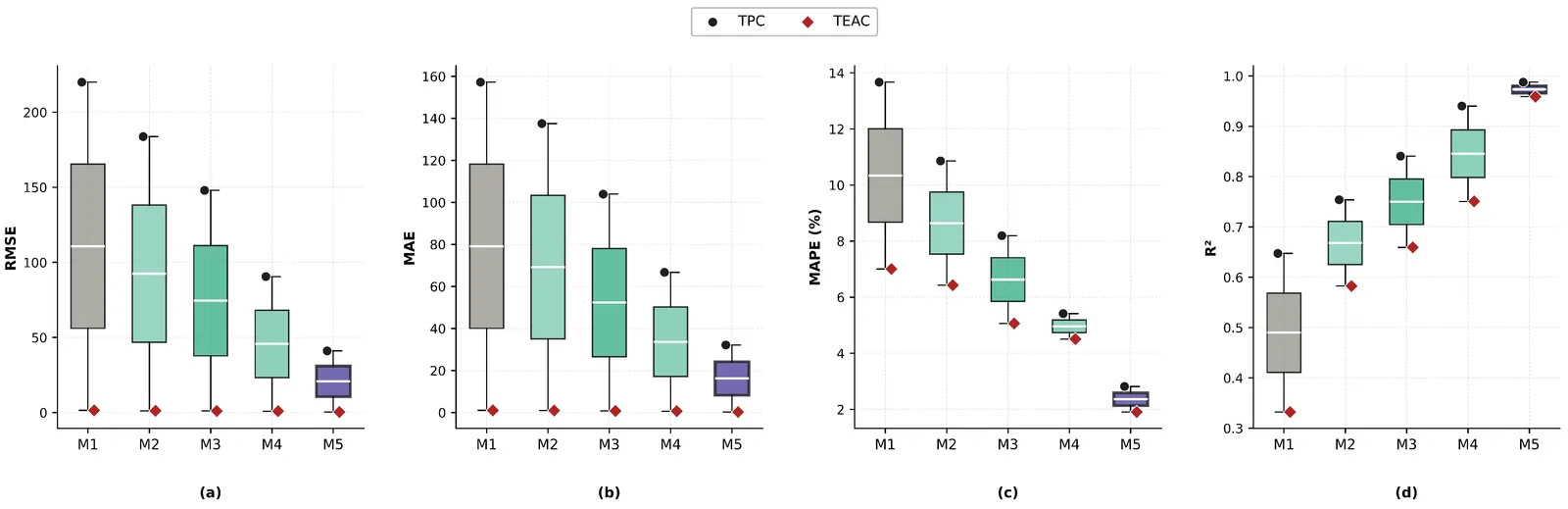
Distribution of (**a**) RMSE, (**b**) MAE, (**c**) MAPE and (**d**) $R^{2}$ across the five configurations.

The distributional view in Figure 24 shows that the gain is not merely a shift in central tendency. The interquartile range of the error contracts monotonically towards the proposed model, whose box is both the lowest and the tightest for RMSE, MAE, and MAPE, while its *R*^2^ box tightens near unity. The framework is therefore not only more accurate on average but more consistent, and consistency is the property that decides whether a predictive tool can be trusted in routine use rather than merely tabulated in a paper. A model whose error varies widely from sample to sample cannot support a release decision, however favorable its mean performance, because the operator has no way of knowing which sample is which.

**Figure 25:**
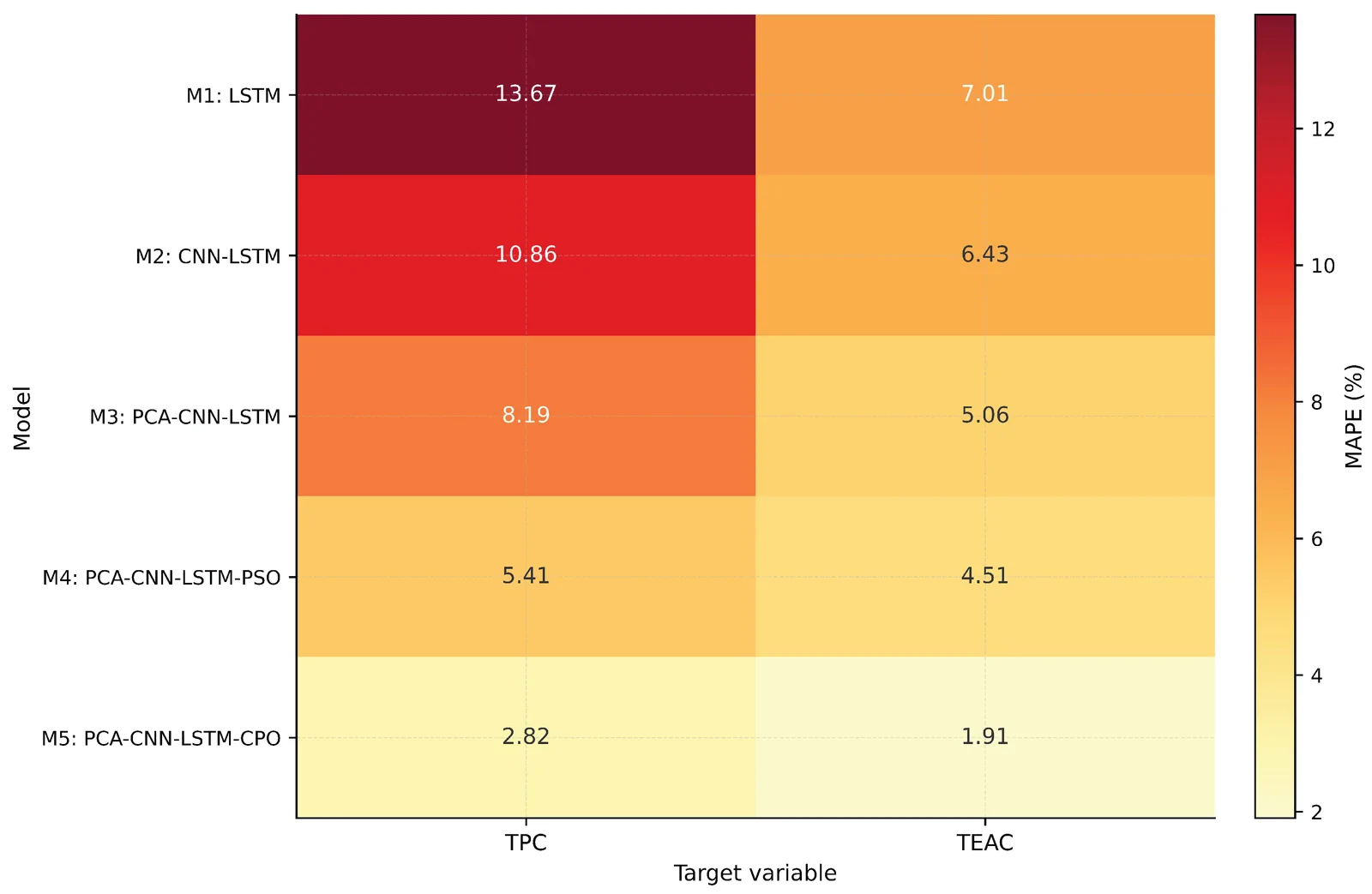
MAPE of the five configurations for both bioactive targets.

Figure 25 condenses the ablation into a single comparison and shows the ordering holding for both endpoints simultaneously, with TEAC consistently the easier target. That asymmetry is coherent with the chemistry, since TEAC spans a narrower relative range and, being an aggregate functional property rather than a sum over chemically diverse species, is buffered against the compositional shifts, notably the proanthocyanidin cleavage discussed earlier, that render TPC the more volatile response.

## 5. Conclusions

The proposed model in this study, PCA–CNN–LSTM–CPO, predicts the TPC and TEAC of grape seed-enriched kefir from ten routine physicochemical, textural, and color descriptors, substituting inference for two destructive assays. Its departure from prevailing food quality models lies upstream of the network. Rather than admitting raw, mutually correlated descriptors and treating each sampling occasion as independent, the framework projects the panel onto an orthogonal chemometric basis that conditions the input space instead of merely compressing it, comprising five components preserving 86% of the variance while dissolving the textural collinearity that would otherwise degrade the conditioning of the input Gram matrix; it then promotes storage day to an explicit temporal axis and masks the terminal targets, recasting shelf-life estimation as a sequence problem in which the trajectory of a sample, not its terminal state, carries the signal. Every fitted transformation is estimated on the training partition alone. Under this protocol the model attained an $R^{2}=0.988$ and a MAPE of 2.82% for TPC, together with an $R^{2}=0.959$ and a MAPE of 1.91% for TEAC, with prediction errors comparable to the replicate dispersion of the reference assays. Reliability was demonstrated rather than inferred from these figures: residuals carried no trend against the predicted value; Shapiro–Wilk did not reject normality for either target, with W = 0.943, p = 0.687 for TPC and W = 0.858, p = 0.220 for TEAC; and Bland–Altman returned a mean difference of −7.33 mg GAE/L, 0.6% of the mean concentration, with limits of agreement free of proportional widening. Absent such drift, the framework substitutes for the assays rather than merely fitting them.

Accuracy of this order from 60 observations is a design outcome. Dimensionality was halved before training; the tensor encoding exploited rather than discarded the dependence among the three time points of each replicate; controlled perturbation quadrupled the training set without eroding between-group contrast; and a median-anchored split confined every test target to the interpolation range. Training and validation trajectories converged without separation. The ablation resolves the individual contributions: convolutional extraction reduced TPC error by 16.5%, chemometric orthogonalization by a further 19.5%, and CPO-based search by 54.7% relative to PSO under an identical architecture, search domain, fitness function, and evaluation budget, with the optimizer as the sole variable. That margin isolates the value of a search that reallocates a contracting evaluation budget toward the promising region, the binding constraint whenever one fitness call costs a full training run. Experimentally, alginate encapsulation delivered roughly 60% more phenolic material than free extract at equal doses, preserved it more effectively through storage, and compressed replicate dispersion from 3.1% to 0.7–1.2%, improving batch consistency alongside retention.

Two limits bound these claims. The observations derive from a single milk source, starter, and production run, so the reported accuracy evidence is interpolation within a characterized design rather than transfer across matrices, and the Folin–Ciocalteu index responds to milk peptides as well as polyphenols. Replicating the design across production batches and other fermented dairy matrices would test transferability, while compound-resolved profiling would refine an aggregate index into specific targets, allowing the framework to predict not only how much antioxidant capacity persists through storage but also which molecules sustain it. Several further boundaries should be acknowledged. The dataset is modest and, despite the leakage-safe protocol, chemometric compression and bounded augmentation used to counter it, a residual risk of overfitting inherent to deep learning on small food datasets cannot be entirely excluded. Formal analytical method validation parameters (limits of detection and quantification, calibration linearity and measurement uncertainty) were outside the present scope, although both bioactive assays are standardized reference methods and replicate coefficients of variation are reported. Finally, no external validation on an independent production run or a different dairy matrix was performed, which we regard as the single most important step before the framework is deployed beyond the characterized design. The present analysis covers a 14-day refrigerated window sampled at three points; because storage day enters the model as an explicit temporal axis, the same architecture can be re-fitted to longer storage once such data become available, and predictive reliability beyond the characterized window is not claimed. The framework could further be extended to predict bioaccessibility by including in vitro digestion endpoints as additional target responses, linking storage retention to the fraction expected to be released under gastrointestinal conditions.

## Figures and Tables

**Table 1:** Experimental kefir formulations used in the study.

Code	Formulation Description
C	Control kefir without grape seed extract addition
FG-1	Kefir containing 1% (*w*/*v*) free black grape seed extract
FG-2	Kefir containing 3% (*w*/*v*) free black grape seed extract
EG-1	Kefir containing 1% (*w*/*v*) alginate-encapsulated black grape seed extract
EG-2	Kefir containing 3% (*w*/*v*) alginate-encapsulated black grape seed extract

**Table 2:** Search space, encoding type and role of the six hyperparameters optimized by CPO and by the particle swarm optimization (PSO) benchmark under an identical fitness function and evaluation budget.

Hyperparameter	Symbol	Domain	Type	Role in the Model
Conv1D filters	f	[16, 96]	Integer	Number of local storage transition feature maps
Kernel size	K	{2, 3}	Integer	Width of the temporal convolution window
LSTM units	h	[32, 128]	Integer	Recurrent memory capacity
Dropout rate	d	[0.10, 0.50]	Continuous	Stochastic regularization
Learning rate	η	[$10^{-4}$, $10^{-2}$]	Log-continuous	Adam update magnitude
Batch size	B	{4, 8, 16, 32}	Discrete	Sequences per gradient update

**Table 3:** Cumulative ablation design for assessing the incremental contributions of model components and metaheuristic optimization strategies.

ID	Model	PCA	CNN	LSTM	Optimizer/Role
M1	LSTM	—	—	✓	Default/recurrent baseline
M2	CNN–LSTM	—	✓	✓	Default/adds local feature extraction
M3	PCA–CNN–LSTM	✓	✓	✓	Default/adds chemometric orthogonalization
M4	PCA–CNN–LSTM–PSO	✓	✓	✓	PSO/classical swarm tuning
M5	PCA–CNN–LSTM–CPO	✓	✓	✓	CPO/proposed model

**Table 4:** Test-set performance of the CPO-optimized PCA–CNN–LSTM model.

Target	RMSE	MAE	MAPE (%)	$R^{2}$
TPC (mg GAE/L)	41.04	32.14	2.82	0.988
TEAC (mM)	0.352	0.275	1.91	0.959

**Table 5:** Ablation results on the held-out test set for both bioactive targets.

		TPC (mg GAE/L)				TEAC (mM)			
ID	Model	RMSE	MAE	MAPE	$R^{2}$	RMSE	MAE	MAPE	$R^{2}$
M1	LSTM	219.97	157.21	13.67	0.647	1.418	1.028	7.01	0.332
M2	CNN–LSTM	183.73	137.51	10.86	0.754	1.121	0.951	6.43	0.583
M3	PCA–CNN–LSTM	147.87	103.91	8.19	0.841	1.013	0.758	5.06	0.659
M4	PCA–CNN–LSTM–PSO	90.51	66.74	5.42	0.940	0.866	0.651	4.51	0.751
M5	PCA–CNN–LSTM–CPO	**41.04**	**32.14**	**2.82**	**0.988**	**0.352**	**0.275**	**1.91**	**0.959**

## Data Availability

The original contributions presented in the study are included in the article/Supplementary Material, further inquiries can be directed to the corresponding author.

## Supplementary Materials

The following supporting information can be downloaded at: https://www.mdpi.com/article/10.3390/foods15162858/s1. Dataset_S1: the complete experimental dataset of 60 observations from five kefir formulations across three storage days with four replicates each, containing all physicochemical, textural, color and bioactive variables used for modelling.

## Abbreviations

The following abbreviations are used in this manuscript:

$a^{*}$	Redness-greenness
ANOVA	Analysis of variance
$b^{*}$	Yellowness-blueness
BN	Batch normalization
BSE	Backscattered-electron detector
C	Control kefir/Unsupplemented control
CNN	Convolutional neural network
CPO	Crested porcupine optimizer
EE	Encapsulation efficiency
EG-1	Encapsulated extract at 1%
EG-2	Encapsulated extract at 3%
FG-1	Free extract at 1%
FG-2	Free extract at 3%
GAE	Gallic acid equivalents
IoT	Internet of things
ID	Identifier
$L^{*}$	Lightness
LC	Loading capacity
LoA	Limits of agreement
LSTM	Long short-term memory
MAE	Mean absolute error
MAPE	Mean absolute percentage error
PBS	Phosphate-buffered saline
PCA	Principal component analysis
PSO	Particle swarm optimization
RMSE	Root mean square error
SD	Standard deviation
SEM	Scanning electron microscopy
SP	Surface phenolic fraction
TA	Titratable acidity
TE	Trolox equivalents
TEAC	Trolox-equivalent antioxidant capacity
TP	Total phenolics
TPC	Total phenolic content
UHT	Ultra-high-temperature
UV–Vis	Ultraviolet–visible
*Vitis vinifera* L.	Black grape seed
WHC	Water-holding capacity
